# Heterogeneous Covalent Organic Framework Membranes Mediated by Polycations for Efficient Ions Separation

**DOI:** 10.1002/advs.202405539

**Published:** 2024-10-30

**Authors:** Shuting Xu, Haibo Lin, Guiliang Li, Qiu Han, Jianqiang Wang, Fu Liu

**Affiliations:** ^1^ Zhejiang International Joint Laboratory of Advanced Membrane Materials & Processes Ningbo Institute of Materials Technology & Engineering Chinese Academy of Sciences Ningbo 315201 China; ^2^ Ningbo College of Materials Technology & Engineering University of Chinese Academy of Sciences Beijing 100049 China

**Keywords:** angstrom channels, covalent organic frameworks, heterostructures, ion sieving, transport mechanisms

## Abstract

Precise ions sieving at angstrom‐scale is gaining tremendous attention thanks to its significant impact at the water‐energy nexus. Herein, a novel polycation‐modulated interfacial polymerization (IP) strategy is developed to prepare a heterogeneously charged covalent organic frameworks (COFs) membrane. Cationic poly(diallyldimethylammonium chloride) (PDDA) regulates the growth and assembly of anionic COFs nanosheets, which thus provides a negative, smooth top surface and positive, rough bottom surface, indicating the presence of heterogeneously charged angstrom‐scale channels through the membrane. Experiments and simulations are conducted to understand the facilitated ions transport behavior relative to specific interactions raised by heterogeneously charged channels and angstrom‐scale steric hinderance as well, rendering the membrane with robust mono‐/divalent cations sieving capabilities. The selectivity (61.6) of Li^+^ to Mg^2+^ in mixed saline under the continuous cross‐flow filtration mode is superior to most of the reported nanofiltration membranes. This polycation‐mediated interfacial polymerization strategy offers a compelling opportunity to develop versatile heterogeneously charged COF membranes for exquisite ion sieving.

## Graphical abstract

A polycation‐assisted interfacial polymerization strategy to synthesize heterogeneous COF (TpPa‐SO_3_H) membrane. It displays the asymmetric charge distribution from negatively charged entrance, weakly charged inside to positively charged exit, respectively corresponding to the instant reception, fast migration, and selective discharge of cations. The prepared dense and surface defect‐free membrane can realize excellent Li^+^/Mg^2+^ separation under crossflow operation.

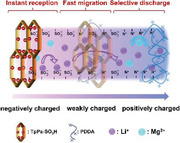

## 1 Introduction

The precise separation of mono‐/divalent ions holds significant implications for the reclamation of valuable resources, e.g. the extraction of lithium from salt lakes.^[^ ^1^ ^]^ However, it presents a formidable challenge for ion sieving in view of the slight disparities in hydrated radii and physicochemical properties between monovalent and divalent ions.^[^ ^2^ ^]^ Pressure‐driven nanofiltration membranes can achieve continuous water‐salt separation. The pore size of nanofiltration membranes typically ranges from 0.5 to 2 nm.^[^ ^3^ ^]^ Traditional polyamide nanofiltration membranes with piperazine and trimesoylchloride as reaction monomers can realize mono‐/divalent ions separation mainly based on size exclusion and the Donnan effect.^[^ ^4^ ^]^ However, the inherently amorphous crosslinking network of polyamide gives rise to a “trade‐off” effect between selectivity and permeability.^[^ ^5^ ^]^

Emerging 2D porous materials exhibit well‐defined, orderly aperture and tailored functionality, which are expected to enhance ion selectivity.^[^ ^6^ ^]^ Covalent organic frameworks (COFs) are organic crystalline materials connected by covalent bonds of light elements (C, N, O, B etc.).^[^ ^7^ ^]^ It is flexible to design the geometry structure and surface properties of channels through selecting diverse molecular building blocks.^[^ ^8^ ^]^ Highly crystalline and insoluble COFs can be first synthesized by hydrothermal method and then processed into membranes via top‐down, e.g., vacuum filtration, which limits its scale‐up preparation and robust operation.^[^ ^9^ ^]^ Interfacial polymerization (IP) is widely applied to continuously prepare polyamide membranes in industry. Crystalline COFs membranes with intrinsic microporosity can be also prepared via the interfacial reaction of molecular building blocks.^[^ ^10^ ^]^ Besides, IP facilitates the preparation of self‐standing and transferable membranes on a large scale.^[^ ^11^ ^]^ Nevertheless, the weak van der Waals interaction between layers makes self‐standing COFs membranes difficult to withstand practical cross‐flow shearing under hydraulic pressure.^[^ ^12^ ^]^ Therefore, it is particularly significant to balance the rigidity of COFs nanocrystals with the flexibility of polymeric membranes to withstand cross‐flow filtration rather than static diffusion and dead‐end filtration.

Another crucial concern is that the nanoscale pore size of COFs membranes fails to achieve precise ions sieving. The channel size of common COFs lies at 1–5 nm, larger than the hydrated ions (0.65–0.8 nm). Combining polymers with COFs can improve membrane selectivity, integrity, and strength as well.^[^ ^13^ ^]^ Cellulose nanofibers^[^ ^14^ ^]^ or dopamine^[^ ^15^ ^]^ have been utilized to enhance the interlamellar interaction of COFs and shrink the channel size to match the target small molecules or ions. In addition to size exclusion, the binding affinity and charged effect can also manipulate the ion‐selective separation.^[^ ^2^, ^16^ ^]^ Notable efforts have been made to introduce specific binding sites or ionic groups into the COFs framework to improve the ions selectivity usually under osmotic diffusion manner.^[^ ^8^, ^17^ ^]^ To the best of our knowledge, this is the first report of COF membranes with asymmetric charged angstrom channels to enhance ions sieving under cross‐flow filtration.

Herein, we report a polycation‐modulated IP strategy to synthesize a heterogeneously charged membrane by manipulating the asymmetric distribution of polycation in sulfonated COFs membrane. Flexible polycation macromolecules Poly(diallyldimethylammonium chloride) (PDDA) with high positive charge density present in the aqueous phase can promote growth and assembly of sulfonated COFs nanosheets during the interfacial polymerization process and eliminate the boundary cracks of the membrane by “adhesive tape” effect. The flexible long chain of PDDA entangled with sulfonated COFs nanosheets is able to tailor the membrane with a negatively charged top surface and positively charged bottom surface, meanwhile modulating the channel size to angstrom scale. The polycation‐mediated COFs membrane (PCOF membrane) shows exquisite mono‐/divalent cations selective separation and robust operational stability under cross‐flow filtration. The sufficient experiments and simulations reveal the instant reception, fast migration, and selective discharge of ions from the negative entrance, and weakly charged intersection to the positive exit, respectively. This study offers a promising strategy for finely modulating the ionic COFs membranes for selective ion separation.

## 2 Results and Discussion

### 2.1 Polycation‐Modulated IP for PCOF Membrane

**Figure 1:**
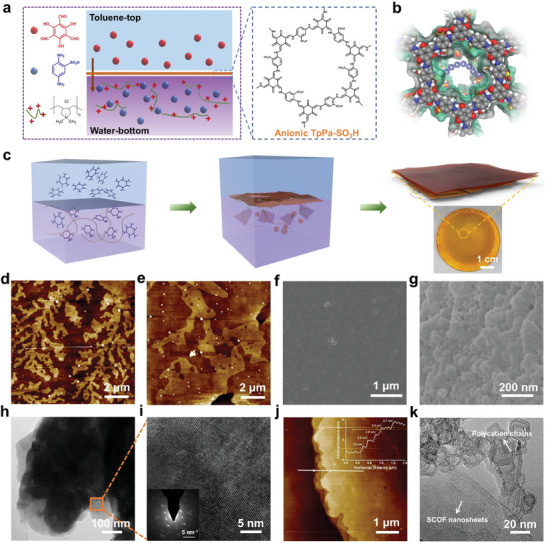
Preparation process and structures of the PCOF membrane. a) Schematic illustration of liquid‐liquid IP for synthesizing the PCOF membrane and chemical structure of the SCOF framework. b) Top view of heterogeneous structure of the PCOF framework. c) Schematic showing the formation process of the PCOF membrane. d) and e) AFM characterization of SCOF nanosheets with different morphology captured from the aqueous solution with a reaction time of 6 h. SEM images of f) top and g) bottom morphology of the PCOF membrane. h) TEM image of PCOF nanosheets in the aqueous phase. i) HRTEM image of selected area of thin SCOF nanosheets. The insert is SAED. j) AFM characterization of the large‐scale SCOF nanosheets after ethanol ultrasonic stripping with the PCOF membrane. k) TEM characterization of PDDA chains conformation, which are curled, aggregated, and entwined at the edge of SCOF nanosheets.

We introduced polycation (PDDA) into the aqueous phase during the IP process of sulfonated COFs to prepare PCOF membrane (**Figure** **1**a). β‐ketoenamine TpPa‐SO_3_H (SCOF) was synthesized as the membrane skeleton, incorporating negatively charged moieties by using 2,4,6‐trihydroxy‐1,3,5‐benzenetricarbaldehyde (Tp) and 2,5‐diaminobenzenesulfonic acid (Pa‐SO_3_H) as the reactive monomers. PDDA was entangled with SCOF nanosheets mainly in the lower part of the membrane to form the heterogeneously charged structure (Figure 1b). The evolution process of the PCOF membrane is depicted in Figure 1c, showing the monomer diffusion, nanosheets growth, and assembly at the immiscible oil/water interface. An intact PCOF membrane deposited on the PTFE substrate was prepared in a diameter of 5 cm within 6 h, showing its productivity and potential for scaled up.

Tp monomers diffused from the organic phase to the aqueous phase containing Pa‐SO_3_H and PDDA to trigger the reversible Schiff base reaction, then generating stable β‐ketoenamine‐based COFs via irreversible tautomerization. We were able to capture the dendritic and integral structure of SCOF nanosheets present in the aqueous phase during interfacial reaction by atomic force microscope (AFM) characterization (Figure 1d,e), indicating the probable nucleation and growth processes of SCOF nanosheets. The long‐chain polycation with abundant positive charges acts as the “tree trunk” template and provides nucleation sites to attract negatively charged Pa‐SO_3_H for further reaction. With the reaction progressed, the branched structure tended to coalesce into large and intact nanosheets (Figure S1, Supporting Information). The generated PCOF membrane displayed a smooth and intact top surface and a rough bottom surface rugged with relatively small‐scale nanosheets aggregates (Figure 1f,g). It is thought that the flexible long chain of PDDA acts as “adhesive tap” to connect adjacent SCOF nanosheets of diverse dimensions and qualities by electrostatic interaction, consequently reducing stress concentration and eliminating the grain‐boundary defects. Thus, the introduction of PDDA is conducive to the robust and large‐scale assembly process of COFs membranes at the interface (Figure S2, Supporting Information). The resultant membrane thickness was ≈100 nm and the surface roughness was as low as several nanometers (Figures S3 and S4, Supporting Information). Comparatively, the SCOF membrane without PDDA showed apparent defects or cracks from scanning electron microscopy (SEM) images despite its higher thickness (Figure S5, Supporting Information).

The transmission electron microscope (TEM) image showed the laminar stack of SCOF nanosheets after removing PDDA with ethanol (Figure 1h). Through high‐resolution TEM (HRTEM), we visually observed the lattice fringe images with independent diffraction direction (Figure 1i), indicating the orderly arrangement structure and high crystallinity of SCOF nanosheets. The lattice fringe distance was ≈3.5 Å, in accordance with the theoretical interlamellar spacing of SCOF. The selected‐area electron diffraction (SAED) displayed sparkling spots, further confirming the high crystallinity of synthesized SCOF nanosheets (inset of Figure 1i). The intercalation of PDDA and also the presence of inhomogeneous SCOF nano‐aggregates caused the less regular laminar stacking of SCOF nanosheets, which is reflected by the relatively weak diffraction peak of (100) crystal plane and broad diffraction peak of (001) located at 27.6° by small angle X‐ray scattering (SAXS) characterization (Figure S6, Supporting Information). Through ethanol ultrasonic treatment, the PCOF membrane can be stripped into SCOF nanosheets with a thickness of around 3 nm (Figure 1j), and the TEM image showcased that PDDA chains were curled, aggregated, and entwined mainly at the edge of SCOF nanosheets under the action of external force (Figure 1k; Figure S7, Supporting Information). It is therefore inferred that PDDA binds between the edges or interlayers of SCOF nanosheets relying on electrostatic attraction to form amorphous structure and generate polycation‐dominated transport channels. Thermal gravimetric analyzer (TG) also demonstrated the presence of two distinct components in the PCOF membrane, ascribed to SCOF and PDDA respectively (Figure S8, Supporting Information).

We further utilized UV‐vis spectra, dynamic light scattering (DLS), and zeta potential characterizations to understand the fundamental reaction process of polycation‐modulated IP. The formation of SCOF units can be determined by monitoring the UV absorption peak of the aqueous phase at 2, 4, and 6 h. As for the PCOF aqueous phase containing PDDA, a new peak with higher absorption wavelength and intensity emerged at 454 nm and gradually intensified with reaction time compared with the SCOF counterpart, verifying the generation of a larger conjugated structure and faster reaction rate (Figure S9, Supporting Information). The darker color of PCOF aqueous solution with the extension of reaction time and Tyndall effect both supported the faster progress of interfacial reaction (Figure S10, Supporting Information). Besides, the average hydrodynamic size of PCOF aqueous phase decreased from 1094 to 160 nm, indicating that PDDA was entangled with sulfonated COF nanosheets to form the membrane rather than being dispersed in the aqueous solution (Figure S11, Supporting Information). Moreover, the zeta potential of PCOF aqueous phase maintained the positive value of ≈+50 mV compared with SCOF solution (≈‐20 mV), implying that the long‐chain polycation tends to shield negative charges of Pa‐SO_3_H by electrostatic interaction (Figure S12, Supporting Information).

The influences of PDDA with diverse molecular weights (Mw) on PCOF membrane were investigated. Close inspection of surface morphologies and preliminary desalination tests manifested that the PCOF membrane with PDDA 400–500k held the most compact morphology (Figures S13 and S14, Supporting Information), comparing to SCOF, PCOF with PDDA<100k, and PCOF with PDDA 100–200k. Microscopic infrared spectrometer (Micro‐FTIR) verified the formation of SCOF components with different molecular weights of PDDA. The wavenumbers of sulfonic acid groups with a little red shift demonstrated the electrostatic interaction between the sulfonic acid groups and quaternary ammonium nitrogen atoms (Figure S15, Supporting Information). Besides, the assembly of the PCOF membrane was substantially shortened to 6 h for achieving a desirable membrane for desalination compared with the traditional IP that needs several days to obtain a self‐standing COF membrane (Figure S16, Supporting Information).

### 2.2 Heterogeneously Charged Channel

The micro‐structure of the channel mainly determines the ions transport behavior through the membrane. The intercalated polycation plays a crucial role in regulating the heterogeneous structure of PCOF membrane channels. X‐ray photoelectron spectroscopy (XPS) survey spectra clearly showed the discrepant chemical compositions between the top and bottom surface of PCOF membrane (Figure S17, Supporting Information). The O 1s spectrum displayed that the SO_3_H group content of top layer (53.1%) was much higher than that of the bottom layer (35.7%). Meanwhile, in the bottom of the PCOF membrane, the content of C─C═N (present in SCOF) was lower and the content of R─N^+^ (present in PDDA) was higher than the top layer, implying that more PDDA chains were enriched in the bottom of PCOF membrane. The element mass/atomic fractions of both sides of the PCOF membrane also elucidated more S elements lie on the upper part and more N elements were present on the lower part (Figure S18 and Table S1, Supporting Information).

To better understand the heterogeneous charge distribution along the transport channel of PCOF membrane, an XPS depth profile for N 1s from the top to the bottom of the PCOF membrane was conducted with etch time to analyze the variations of peak area of R‐N^+^ and C═C─N. It took 90 s to finish the etch process vertically through the PCOF membrane with a thickness of ≈100 nm. It is noted that the etch time does not relate to the etch depth linearly due to the inhomogeneous structure of the PCOF membrane. We therefore cannot acquire the charge distribution along the channels accurately. Nevertheless, we are still able to get a glimpse of the asymmetric distribution of negative and positive charges along the channel inside the membrane.

**Figure 2:**
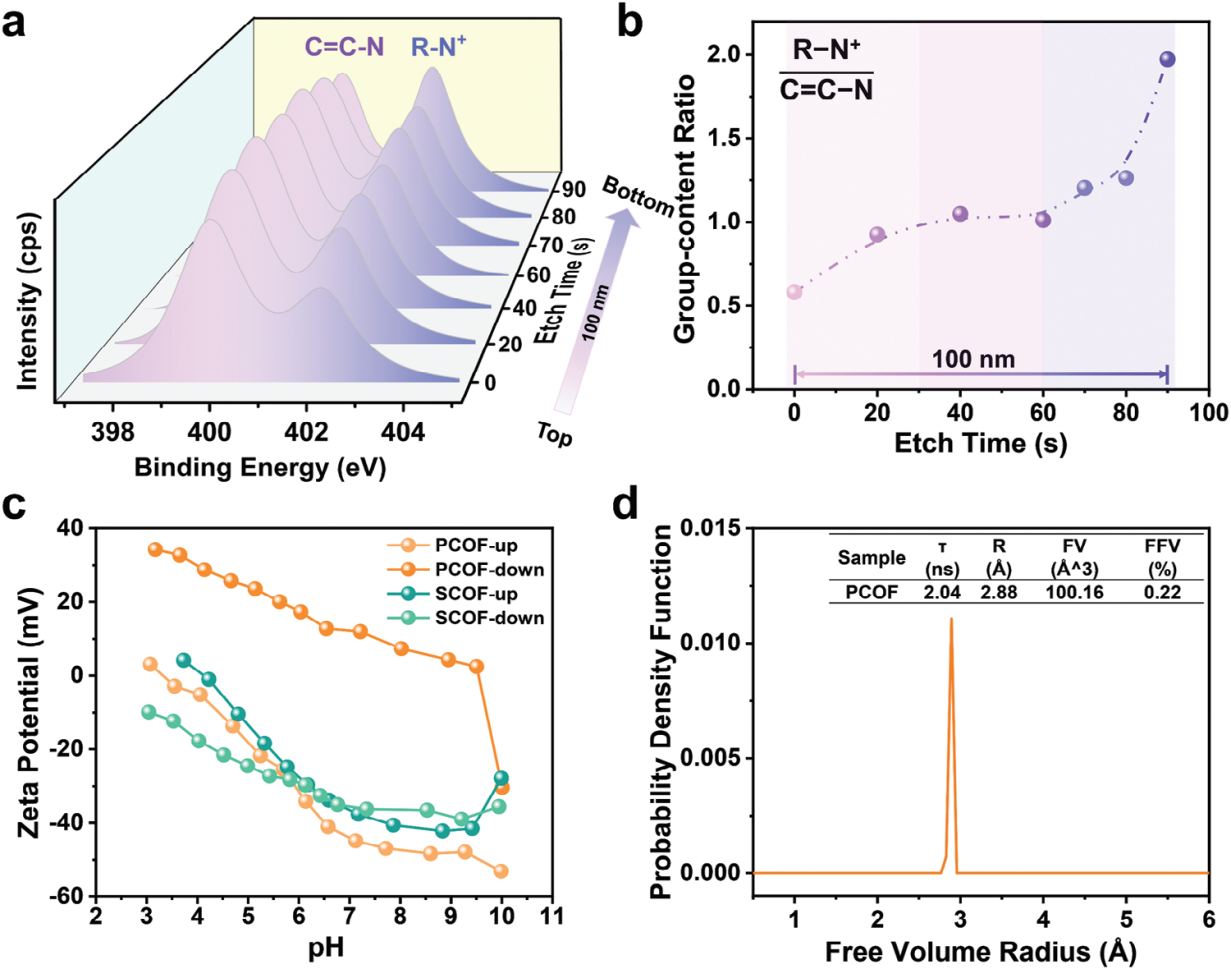
Heterogeneously charged distribution in the angstrom‐scale transport channel. a) XPS depth profile with N 1s of the PCOF membrane from the top to the bottom. b) Ratio variation of R‐N^+^ to C═C─N with the increasing etch time. c) Solid surface zeta potentials of both sides of the SCOF membrane and the PCOF membrane. d) PALS characterization of the PCOF membrane, confirming the average free volume radius and the fractional free volume.

As depicted in **Figure** **2**a,b, the ratio of R‐N^+^ to C═C─N slightly increased in the initial etch time of 30 s, and then kept almost constant between 30 and 60 s, and finally rose precipitously when the etch time was beyond 60 s. Moreover, the solid surface zeta potential demonstrated the oppositely charged surfaces of the PCOF membrane in contrast to the homogeneously negatively charged SCOF membrane. The top surface approaching to the organic phase exhibited negative potential with the isoelectric point of 3.32, but the back surface close to the aqueous phase displayed high positive potential with the isoelectric point of 9.54 (Figure 2c). It is thus inferred that positively charged PDDA chains are mainly entangled with SCOF aggregates in the lower part of the membrane because of the diffuse entropy barrier and strong affinity to the water phase, and the upper part is mainly composed of negatively charged crystalline SCOF nanosheets. The middle part is weakly charged due to the balance of R‐N^+^ and ‐SO_3_^−^ groups. Moreover, the prominent difference of zeta potential between the top and bottom surface of the PCOF membrane indicated a build‐in micro‐electric field in the membrane. Especially at pH = 7, the zeta potential difference was as high as 60, much higher than the counterparts at pH = 3 and 10. The zeta potential difference between both surfaces dropped to ≈20 at pH = 10. The Positron Annihilation Lifetime Spectroscopy (PALS) was implemented to calculate the lifetime of orthoPositroni‐um (oPs) and determined the average free volume radii (≈2.88 Å) of PCOF membrane, and the fractional free volume (FFV) of 0.22% (Figure 2d; Figure S19, Supporting Information). PALS results validated that the interaction between PDDA flexible chain segments and SCOF skeleton units synergistically constructed the angstrom‐scale transport channel. The contractible angstrom channels are mainly attributed to the occupation of polycation chains between the edges and interlayers of SCOF nanosheets, and the interplay between crystalline SCOFs and amorphous β‐ketoenamine‐based polymers (Figure S20, Supporting Information). It verifies that the polycation‐mediated IP offers a facile way to prepare anionic COFs membranes with angstrom channels.

### 2.3 Precise Ion Sieving and Excellent Desalination Performances

**Figure 3:**
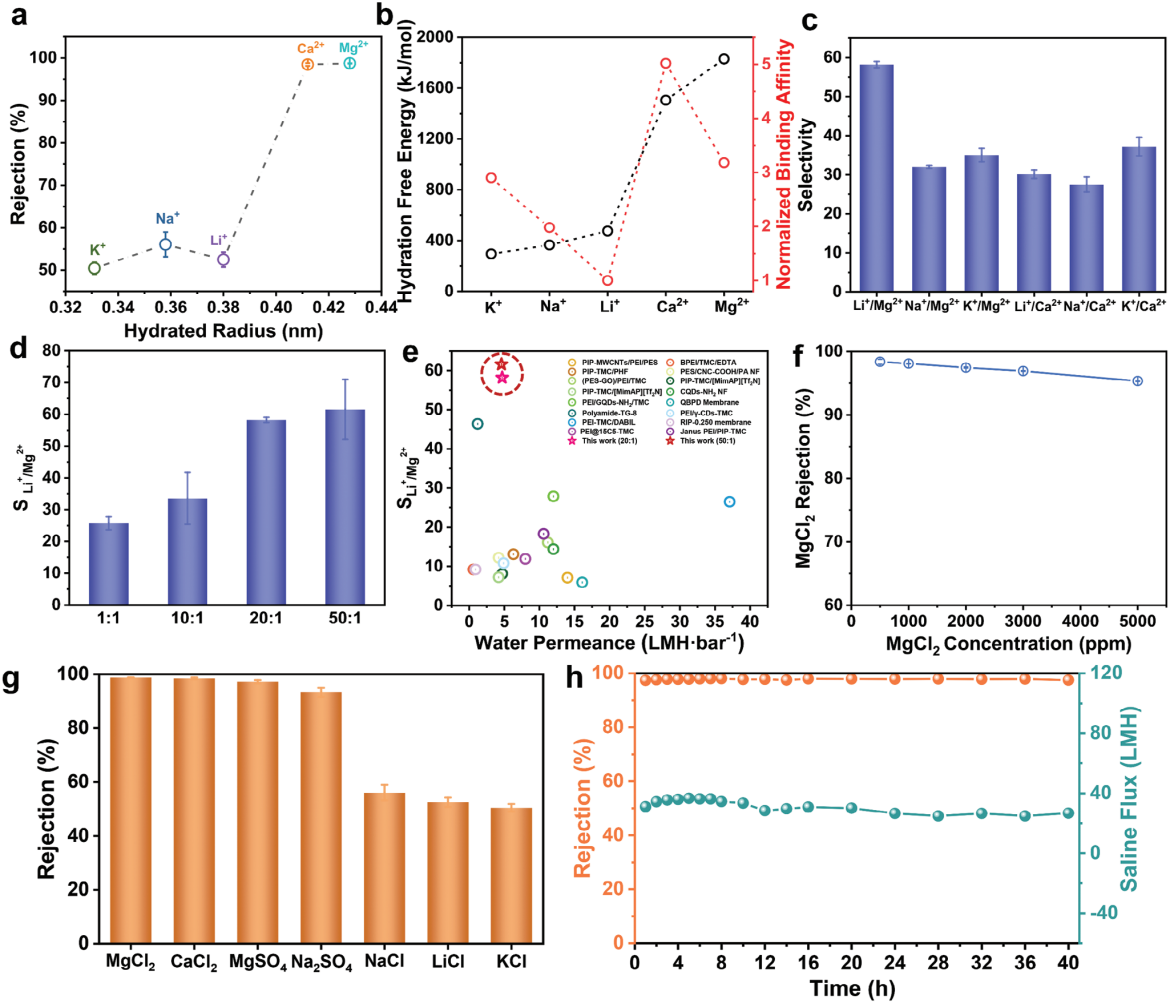
Cation sieving and desalination performances by the PCOF membrane. a) Rejection of a series of mono‐/divalent cations with different hydrated radii. b) Hydration‐free energy of divergent ions and normalized binding affinity of mono‐/divalent cation with sulfonate group. c) A series of mono‐/divalent cation selectivity. d) Li^+^/Mg^2+^ selectivity with diverse concentration ratios of MgCl_2_ to LiCl. e) Comparison of Li^+^/Mg^2+^ selectivity between polymer‐based nanofiltration membrane and the PCOF membrane under similar cross‐flow filtration by pressure. f) Rejection of MgCl_2_ with increasing concentration of feed solution from 500 to 5000 ppm. g) Capabilities of single‐salt rejection. h) Long‐term operational stability under cross‐flow conditions at 6 bar.

Steric exclusion, Donnan exclusion, and binding affinity are the dominant mechanisms for ions sieving in the confined nano‐channels.^[^ ^1^, ^2^, ^8^, ^18^ ^]^ We selected several divalent and monovalent cations to evaluate desalination performances using the PCOF membrane mediated by PDDA 400–500 k. These cations are in the form of hydrated ones in water, holding sub‐angstrom difference with hydrated radii of Mg^2+^, Ca^2+^, Li^+^, Na^+^, and K^+^ of 4.28, 4.21, 3.80, 3.58, and 3.31 Å. The hydrated ions would experience partial dehydration so as to be squeezed into the entrance of the confined Angstrom channels.^[^ ^19^ ^]^ The hydration energy is usually positively related to ion size. Ions with small hydrated shells with low hydrated energy are easier to remove their surrounding water to partition into channel mouth.^[^ ^2^, ^20^ ^]^ The ions interception sequence is Mg^2+^ > Ca^2+^ > Na^+^ > Li^+^ > K^+^, which is directly correlated to the hydration energy of ions except for Li^+^ (**Figure** **3**a). After partition into the entrance, Li^+^ traverses the channel with less diffusion friction than Na^+^ because of the discrepancy of binding affinity to sulfonate groups on the channel walls. Normalizing the binding affinity of diverse ions as Li^+^, we observed that Li^+^ possesses the lowest binding affinity to the sulfonate group, which promotes the easier dissociation and hopping from one affinity site to the adjacent one (Figure 3b).^[^ ^21^ ^]^

In order to further evaluate the ions sieving capability of PCOF membranes, especially for Li^+^ to Mg^2+^ selectivity, which plays an extremely pivotal role in recovery of lithium resources from salt lakes.^[^ ^22^ ^]^ We utilized the PCOF membrane to filtrate the mixed cations solution in a continuous cross‐flow manner under 6 bar rather than the low efficient batch diffusion commonly operated in previous studies. The ratio of divalent to monovalent salt in the feed solution was set as 20:1 (Figure S21, Supporting Information). Because of the faster transport of Li^+^, the Li^+^/Mg^2+^ selectivity reached an optimal value of 58.2. Other mixed solution similarly exhibited desirable mono‐/divalent ion selectivity, which was almost above 30 (Figure 3c). Moreover, the Li^+^/Mg^2+^ selectivity increased with increasing the ratio of MgCl_2_ to LiCl from 1:1 to 50:1 (Figure 3d). In the binary system, the competition effect between ions intensifies in view of the enhanced relative concentration of Mg^2+^, which causes the decrease of Mg^2+^ flux or increase of Li^+^ flux. When the ratio of MgCl_2_ to LiCl was 50:1, the Li^+^/Mg^2+^ selectivity can reach 61.6, which represents the competitive performance compared with the commercial nanofiltration membranes and reported membranes (Figure 3e and Table S2, Supporting Information). The PCOF membrane also demonstrated excellent MgCl_2_ rejection above 95%, even with the feed solution up to 5000 ppm (Figure 3f).

In addition to sieving the mixed salt solution, a series of single salts were also applied to cross‐flow test. The order of rejection was MgCl_2_ (98.8%) > CaCl_2_ (98.5%) > MgSO_4_ (97.2%) > Na_2_SO_4_ (93.3%) > NaCl (56.1%) > LiCl (52.5%) > KCl (50.5%), which validated the superior capabilities of rejecting both divalent cationic and anionic salts due to the special channel with the combination of angstrom‐scale and heterogeneously charged apertures (Figure 3g). In addition, the PCOF membrane showed operational robustness in 40 h under cross‐flow at 6 bar. The rejection of MgCl_2_ maintained 98% and the saline flux reached 37 L m^−2^ h^−1^ (LMH) (Figure 3h). The favorable combination of rigid SCOF nanosheets and flexible polycation chains endows the PCOF membrane with sufficient mechanical stability against substantial hydraulic pressure and cross‐flow shearing. The desirable flux is attributed to desirable water wettability originated from abundant hydrophilic groups and nanoporous structure (Figure S22, Supporting Information). Therefore, the high‐precision ion sieving, excellent desalination performance, together with the mechanical stability impart the PCOF membrane outstanding potential to tackle practical issues of resource recovery and water purification.

### 2.4 Ion Transport Behavior and Selective Sieving Mechanism

**Figure 4:**
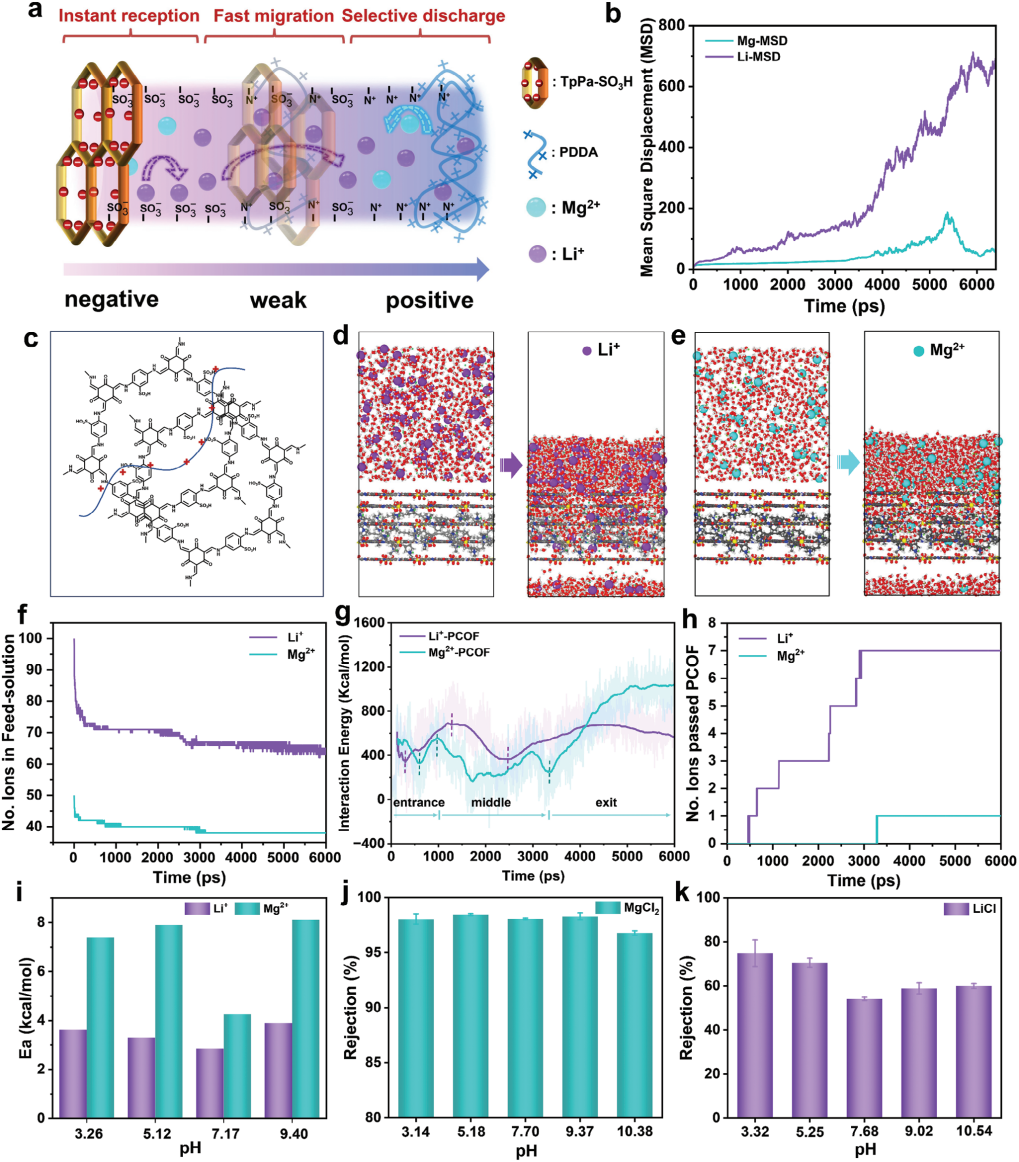
Ion transport and selective separation mechanisms in the charged channel. a) Illustration of ion transport behavior and main mechanism in the channel. b) MSD of Mg^2+^/Li^+^ during the process of MD simulation. c) Molecular structure of the simulation box of PCOF membrane. Screenshots of the dynamic transport process of d) Li^+^ and e) Mg^2+^ by MD simulation from the inlet to the outlet. f) Variation in the number of Mg^2+^/Li^+^ ions in feed solution during the process of MD simulation. g) Interaction energy between Li^+^ or Mg^2+^ and the PCOF framework. h) Ion numbers passing the PCOF model during the process of MD simulation. i) Energy barriers of ions through the angstrom‐scale charged channel at diverse pH values. Rejections of j) MgCl_2_ and k) LiCl solution with different pH values under cross‐flow conditions.

Apart from size exclusion and affinity groups, we speculate that the outstanding Li^+^/Mg^2+^ selectivity is contributed by the charge heterogeneous distribution across the angstrom transport channel. The ion transport channel was decoupled into three parts including entrance, inner part, and exit, as depicted in **Figure** **4**a, and the corresponding roles were elucidated by the theoretical simulation and experiments. Classic molecular dynamics (MD) simulations were performed to reveal the transport behaviors of ions through the PCOF membrane. The mean square displacement (MSD) described the relative motion displacement of ions, which illustrated that Li^+^ achieved faster and easier transmission than Mg^2+^ during the process of simulation (Figure 4b). The construction of the model was consistent with the heterostructure of the PCOF membrane as shown in Figure 4c; Figure S23, Supporting Information. The simulation box was divided into three parts displayed transition from negative charge, weak charge to positive charge. The MD results demonstrated that most of Mg^2+^ are intercepted and Li^+^ can permeate (Figure 4d,e). The entrance with strong negative charges acts as “a cation receptor”, which is conducive to capturing cations into the channel entrance to obtain the initial ions discrimination. As shown in Figure 4f, at the initial short simulation time t < 500 ps, the ion number of Li^+^ in the feed solution rapidly dropped. Meanwhile, the interaction energy between Li^+^ and PCOF channel obviously declined, illustrating the energy barrier of ion entering the channel was offset by the interaction between Li^+^ and binding sulfonic groups. During 500 ps < t < 1500 ps, the increase of interaction energy came from the enhancement of electrostatic attraction by fixed sulfonic ions. With the further extension of simulation time, Li^+^ transported to the middle part of the channel and the interaction energy declined substantially, mainly because the weakly charged intersection reduced channel wall friction and thus significantly promoted ions fast migration (Figure 4g). Subsequently, due to the loosely compacted and rough structure at the channel exit, the energy barrier varied slightly, and lithium ions were still easy to get out of the channel. Therefore, the transport rate of Li^+^ was faster in the range of 1500–3500 ps over the whole simulation time (Figure 4h). In addition, when t > 3500 ps, Li^+^ in the feed solution was still able to constantly and slowly enter the PCOF channel. In contrast, Mg^2+^ holds larger hydrated ion size and hydration energy, first the ion numbers entering the channel were lower than Li^+^ (Figure 4f). For Mg^2+^ migration in the channel, the higher binding affinity hindered its diffusion. At the exit, the intense electrostatic repulsion between Mg^2+^ and the quaternary ammonium nitrogen of PDDA further inhibited the release of Mg^2+^, confirmed by considerable enhancement of the interaction energy between Mg^2+^ and the PCOF framework, which was 1.8 times than that of Li^+^. We also can observe that Mg^2+^ took longer time (3300 ps) to pass through the channel compared with Li^+^ permeation only at 480 ps. Therefore, the exit finally expressed the selective discharge of Li^+^ and Mg^2+^.

Furthermore, we performed a series of desalination experiments with different pH values that can modulate the degree of protonation of sulfonic groups and the charged intensity of ion channel. We utilized the Arrhenius type equation (Section S5, Supporting Information) to calculate the transmembrane energy barriers (*E*_a_) of Li^+^ and Mg^2+^ by osmotic diffusion manner (Figures S24 and S25, Supporting Information). The energy barriers of Mg^2+^ were much higher than Li^+^, which validated that the transport of divalent Mg^2+^ is significantly hindered in the charged channel compared with monovalent Li^+^. At pH = 3.26, 5.12, the higher *E*_a_ was mainly ascribed to the electrostatic repulsion as the high protonation of sulfonic acid groups and quaternary ammonium groups. At pH = 7.17, both Li^+^ and Mg^2+^ had the minimum *E*_a_ values, which indicated that the nearly neutral micro‐environment in the channel may promote fast ion migration (Figure 4i). At pH = 9.4, the deprotonation degree of sulfonic acid groups deepens and causes higher *E*_a_ due to the electrostatic attraction, which enhances the transport resistance of Li^+^ and Mg^2+^ through the angstrom channel.

When MgCl_2_ and LiCl solutions were conducted by cross‐flow filtration under enough pressure, the external energy input was sufficient for the hydrated ions to realize the dynamic dehydration process. And the charged effects of confined angstrom channel played a dominant role for ions discrimination. For MgCl_2_ solution, the rejection maintained about 98% from pH≈3.14 to pH≈9.37 but occurred slightly decreased in strong base condition (pH = 10.38), because the more dissociated sulfonates increase the intensity of surface negative charge and inevitably promote the absorption and diffusion of Mg^2+^ (Figure 4j). For the LiCl solution, the neutral environment creates the maximum ions permeance, verifying that weakly charged property in the inner part can markedly facilitate Li^+^ migration with less friction. The built‐in electrostatic field generated from the oppositely charged surface promoted the fast transport of Li^+^ from the negative entrance to the positive exit. In addition, the acid condition showed the highest rejection of LiCl due to the high E_a_ caused by the protonation of sulfonates (Figure 4k). Consequently, the integrally multi‐stage charged effects can alter the built‐in micro‐electric field, and therefore accomplish efficient ion sieving via instant reception, fast migration, and selective discharge of diverse ions.

## 3 Conclusion

In summary, we develop a novel polyelectrolyte‐assisted IP strategy to acquire the robust PCOF membrane with heterogeneously charged angstrom channels, showing superior mono‐/divalent cation sieving ability. The polycation PDDA with highly charged density facilitates the nucleation, growth, and assembly process of SCOF nanosheets via flexible long chains entanglement and electrostatic interaction, yielding an intact morphology and efficient production. The PCOF membrane exhibits a transition from negative charge to positive charge along the angstrom channels. According to the synergistic effects related to steric exclusion, electrostatic interaction, and binding affinity, the PCOF membrane exhibits a remarkable selective partition of Li^+^/Mg^2+^ (61.6), which is superior to state‐of‐the‐art polymer‐based nanofiltration membranes. From the long‐term cross‐flow filtration, the rejection of MgCl_2_ maintains stability without obvious attenuation, indicating the robustness and practicality of the PCOF membrane. This strategy opens a new avenue for designing ion‐sieving membranes with heterogeneously charged channels to tackle practical resource and energy issues.

## Conflict of Interest

The authors declare no conflict of interest.

## Supporting information



Supporting Information

## Data Availability

The data that support the findings of this study are available from the corresponding author upon reasonable request.
