# Supplementary material for: Heterogeneous Covalent Organic Framework Membranes Mediated by Polycations for Efficient Ions Separation
Source: Adv Sci (Weinh). 2024 Oct 30;11(48):2405539. doi: 10.1002/advs.202405539 (PMC11672246; doi:10.1002/advs.202405539)
Supplement: Supplementary file 1 — Supporting Information [file ADVS-11-2405539-s001.docx]

Supporting Information

**Heterogeneous Covalent Organic Framework Membranes Mediated by Polycations for Efficient Ions Separation**

Shuting Xu, Haibo Lin, Guiliang Li, Qiu Han, Jianqiang Wang, and Fu Liu*

S. Xu, H. Lin, G. Li, Q. Han, J. Wang, F. Liu

Zhejiang International Joint Laboratory of Advanced Membrane Materials & Processes

Ningbo Institute of Materials Technology & Engineering, Chinese Academy of Sciences

Ningbo 315201, China

S. Xu, H. Lin, G. Li, Q. Han, J. Wang, F. Liu

Ningbo College of Materials Technology & Engineering

University of Chinese Academy of Sciences

Beijing 100049, China

E-mail: fu.liu@nimte.ac.cn

Keywords: covalent organic frameworks, bioinspired channels, heterostructures, ion sieving, transport mechanisms

**Table of contents**

Section S-1: Materials and chemicals 3

Section S-2: Methods 4

Section S-3: Characterization 5

Section S-4: Separation performance evaluation 7

Section S-5: Ion transport energy barriers measurement 8

Section S-6: Figures 9

Section S-7: Tables 36

Section S-8: Supplemental references 37

# Section S-1: Materials and chemicals

2,4,6-trihydroxy-1,3,5-benzenetricarbaldehyde (Tp, 97%) was bought from Zhengzhou Alpha Chemical Co. Ltd. 2,5-diaminobenzenesulfonic acid (Pa-SO_3_H, 99%) was bought from Shanghai Aladdin Biochemical Technology Co., Ltd. Glacial acetic acid and toluene were from Sinopharm Chemical Reagent Co., Ltd (China). The polycation, poly(diallyldimethylammonium chloride) (PDDA, 20 wt%) of different molecule weight, including *M*w=400,000-500,000, *M*w=100,000-200,000, and *M*w<100,000 (35 wt%), were purchased from Shanghai Aladdin Biochemical Technology Co., Ltd. The inorganic salts (MgCl_2_, CaCl_2_, MgSO_4_, Na_2_SO_4_, NaCl, LiCl, and KCl, CP) were from Shanghai Aladdin Biochemical Technology Co., Ltd. All the chemicals were used without further purification.

# Section S-2: Methods

**Preparation of oil and aqueous two-phase solutions**

0.05 g PDDA (*M*w=400,000-500,000, 20 wt%) was solved in 20 mL water, then 0.1 mmol Pa-SO_3_H were added above solution to obtain aqueous phase after sufficient dissolution. 2.4 g acetic acid diluted to 10 mL as the catalyst was dropped into aqueous phase. The oil phase comprised of 0.067 mmol Tp in 10 mL methylbenzene. The SCOF without PDDA, PCOF with PDDA<100k, and PCOF with PDDA 100-200k were respectively prepared without PDDA, with 0.033 g PDDA (*M*w<100,000, 30 wt%), and with 0.05 g PDDA (*M*w=100,000-200,000, 20 wt%) as the aqueous constituent.

**Preparation of the PCOF thin membrane composite membrane (the PCOF membrane)**

The suction bottle was utilized as the device of PCOF interfacial reaction. The operation process was as follows, firstly, the sand core was covered by PTFE membrane and respectively wetted with ethanol and water in order to retain part of water channels that maintained the interfacial stability while was conductive to deposit the PCOF membrane. Secondly, the commercial PTFE microfiltration membrane (diameter, 5 cm) was nicely placed on the sand core and the filter cup was fixed. Then, 5 mL aqueous solution suctioned by pipette was injected on the surface of PTFE membrane, 3 mL Tp solution was whereafter added as the oil phase. Finally, after certain hours, the PCOF membrane at the interface was slowly and carefully deposited on PTFE substrate without any defects under a little negative pressure. Finally, the PCOF membrane was dried under 60 ℃ to remove excess solvent and strengthened the electrostatic crosslinking effect between TpPa-SO_3_H nanosheets and molecular chain of PDDA before all desalination tests.

# Section S-3: Characterization

The morphologies of as-prepared membranes or nanosheets were investigated by scanning electron microscopy (FESEM, S4800, Hitachi) and scanning probe microscope (Dimension ICON AFM, Bruker) on tapping mode. The surface roughness was obtained by software NanoScope Analysis 1.7. The transmission electron microscopy (TEM, Tecnai F20) was used to characterize the crystallinity of SCOF materials and structural composition. EDS mapping was conducted to analyze the elements distribution of both sides of PCOF membrane. Small angle X-ray scattering (SAXS, Xeuss 3.0 UHR) was utilized to analyze the crystallinity of the PCOF membrane. the Positron Annihilation Lifetime Spectroscopy (PALS, DPLS3000) was applied to obtain the free volume and fractional free volume that reflected the properties of pore and inner structure of the PCOF membrane to some extent.

The peaks’ variation of chemical groups was analyzed with the microscopic infrared spectrometer (Micro-FTIR, Cary660+620, Agilent). X-ray photoelectron spectroscopy (XPS, Axis Ultra DLD, Kratos) was applied to chemical components analysis. The variation of R-N^+^/C=C-N ratio with depth from top to bottom of the PCOF membrane was characterized by XPS etch. The etch conditions were 10 kV, Ar1000+. All samples tested with Micro-FTIR and XPS were deposited on silicon wafer. The simultaneous thermal analyzer (TG-DSC, STA 449F3) was utilized to qualitatively demonstrate the presence of two components.

UV-vis spectroscopy was utilized to evaluate the maximum absorption peaks of SCOF units in two aqueous phases. Dynamic light scattering particle size analyzer (Zetasizer Nano ZS) characterized the colloid sizes in PCOF aqueous solution and was also applied to analyze the variations of charged properties both in SCOF aqueous solution and PCOF aqueous solution by changing the test procedure. Zeta potentials of membrane surfaces were evaluated by the electrolyte analyzer (SurPASS Anton Paar, GmbH) with 1 mM KCl solutions as electrolyte solution over a pH value range of 3-10. The hydrophilicity and wettability of different membranes were measured using contact angle goniometer (OCA25, Dataphysics).

The characterization of salt feed solution and filtrate concentration was obtained from electrical conductivity, measured by electrical conductivity meter (DDSJ-308F, Shanghai leici instruments), besides, the concentration of single cation (Mg^2+^, Ca^2+^, Li^+^, Na^+^ and K^+^) in the mixture was analyzed by inductively coupled plasma emission spectrometer (ICP-OES, SPECTRO ARCOS, Germany).

# Section S-4: Separation performance evaluation

The separation performance of as-prepared membranes was evaluated by using pressure-driven crossflow apparatus at room temperature. The effective filtration area of the cell is 3.14 cm^2^. The salt solutions were as the feed solutions and the membranes were pre-compacted at 6 bar for at least 1 h to ensure the stable desalination performance.

The salt rejection (*R*) and permeation flux were conducted with the following equations (1) and (2).

$R=(C_{f}-C_{p})/C_{f}\times100\%$ (1)

$\text{J=V/(At)}$ (2)

Where *C_f_* is the solute concentration in feed and *C_p_* is the solute concentration in permeate. *V* (L) is the volume of collected filtrate, *A* (m^2^) is the effective membrane area, and *t* is the collecting time (h).

The mono-/divalent cations selectivity (*S*) was calculated by equation (3).

$\text{S=(1}\text{-R}_{1}\text{)/(1}\text{-R}_{\text{2}}\text{)}$ (3)

Where *R_1_* and *R_2_* separately represent the rejection of the monovalent ion (Li^+^, Na^+^ or K^+^) and divalent ion (Mg^2+^ or Ca^2+^).

# Section S-5: Ion transport energy barriers measurement

To evaluate the transmembrane energy barrier of the ion, we utilized the concentration gradient-driven diffusion process. One side of the diffusion cell was the mixed salt solution with 0.1 M MgCl_2_ and 0.1 M LiCl solutions and the other side was the pure water. The ion diffusion rate is depended on the variation of temperature. The temperatures were set to 25, 30, 35, 40, and 45 °C, respectively. The energy barriers (*E_a_* (kcal·mol^−1^)) for Li^+^ and Mg^2+^ across the membrane can be calculated using an Arrhenius-type equation (4):

$\ln\text{P}_{\text{i}}\text{=lnα-(}\frac{\text{E}_{\text{a}}}{\text{R}}\text{∙}\frac{\text{1}}{\text{T}}\text{)}$ (4)

where *P_i_* (mol·m^−2^·h^−1^) is the ion permeation rate. *α* is a pre-exponential factor and *R* (1.985×10^−3^ kcal·mol^−1^·K^−1^) is the gas constant, *T* (K) is absolute temperature. An Arrhenius plot is created with 1/*T* as the independent variable and *lnP_i_* as the dependent variable. Then, the slop of a series of obtained points is related to the energy barrier *E_a_* divided by the gas constant *R*.

# Section S-6: Figures


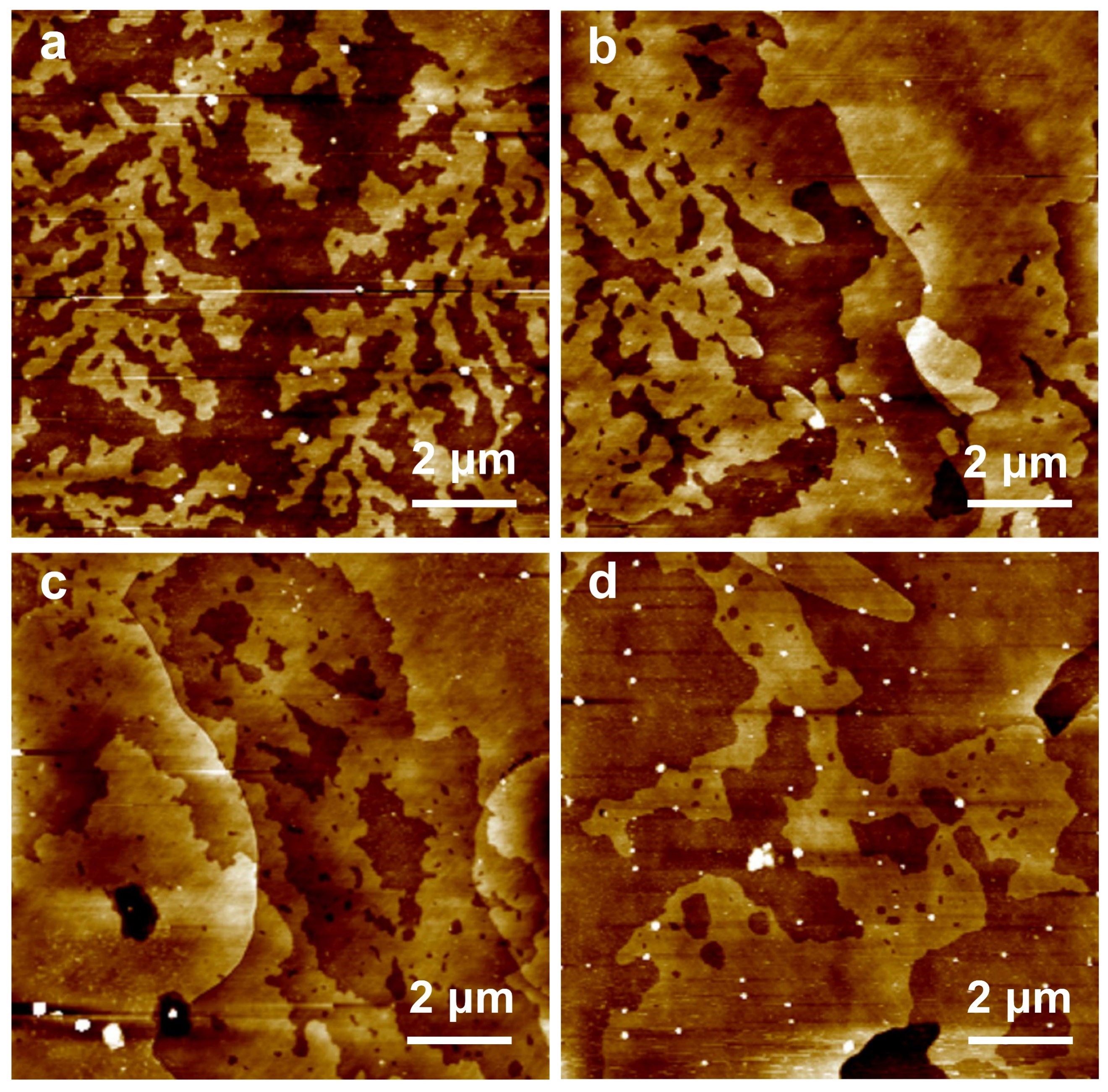

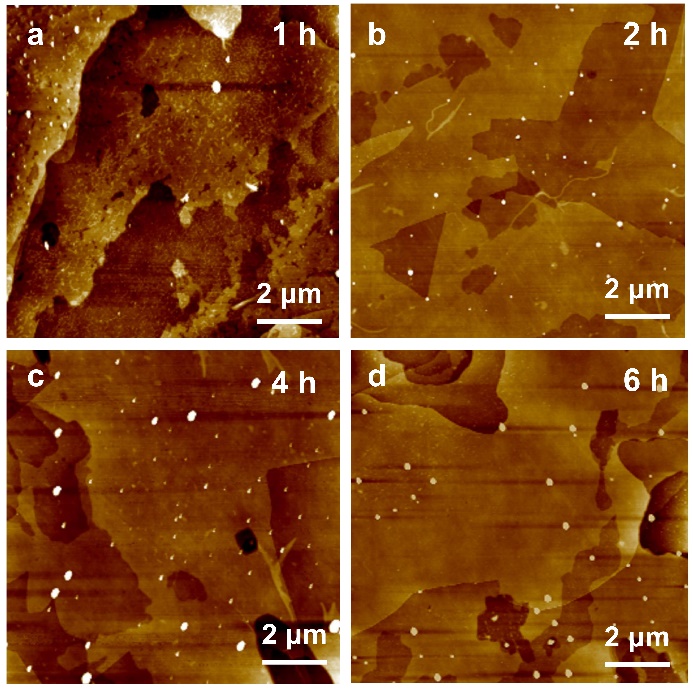


**Figure S1. Left**: AFM characterization of SCOF components with different dendritic and integral structure morphological distribution (a, b, c, d) captured from the aqueous phase of PCOF IP reaction, indicating the nucleation and growth processes. **Right**: AFM morphology characterizations of SCOF nanosheets captured from the aqueous phase of PCOF with the IP reaction a) 1 h, b) 2 h, c) 4h and d) 6h.

**Note:** In order to uncover the variation processes of nucleation and growth of SCOF components, we chose the aqueous solution of PCOF IP reaction to conduct AFM characterization. The aqueous solution was diluted 20 times with deionized water and continued to be diluted 10 times with ethanol before being dripped onto silicon wafer to test. Surprisingly, the branch structure appeared and we comprehend that the molecular chain of polycation with highly positive charge density acts as the template. The charged parts can concentrate amine monomers because of the electrostatic attraction, thus creating amounts of nucleation sites and accelerating the growth of SCOF nanosheets. With the process of reaction deepening, the branches continued to grow and joined into large nanosheets.

To investigate the growth process of SCOF, we further analyzed the growth variation of SCOF components in PCOF aqueous solution with the extension of reaction time (1 h, 2 h, 4 h and 6 h) by AFM characterization. The results showed that the SCOF nanosheets were quickly formed within 1 h. With the extension of reaction time, the SCOF nanosheets continued to grow into relatively bigger and more nanosheets. All the selected solutions were firstly diluted 20 times with deionized water, then are diluted another 10 times with ethanol before being dripped onto a silicon wafer for AFM characterization.


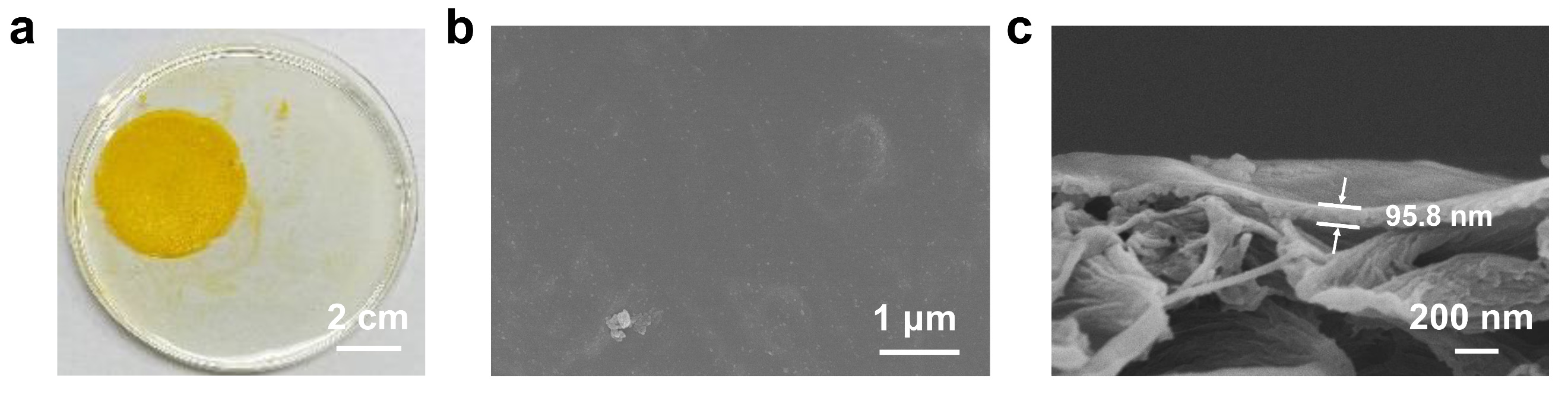


**Figure S2.** (a) PCOF membrane floating on the surface of water. (b) Surface SEM image of the PCOF membrane. (c) Cross-sectional SEM image of the PCOF membrane.

**Note:** The intact and firm PCOF membrane can be self-standing on the surface of water without cracks or deposited on a substrate as the thin membrane composite membrane. From the scanning electron microscopy (SEM) characterization, the surface of PCOF membrane was dense and defect-free and the thickness was about 100 nm from the cross-sectional image.


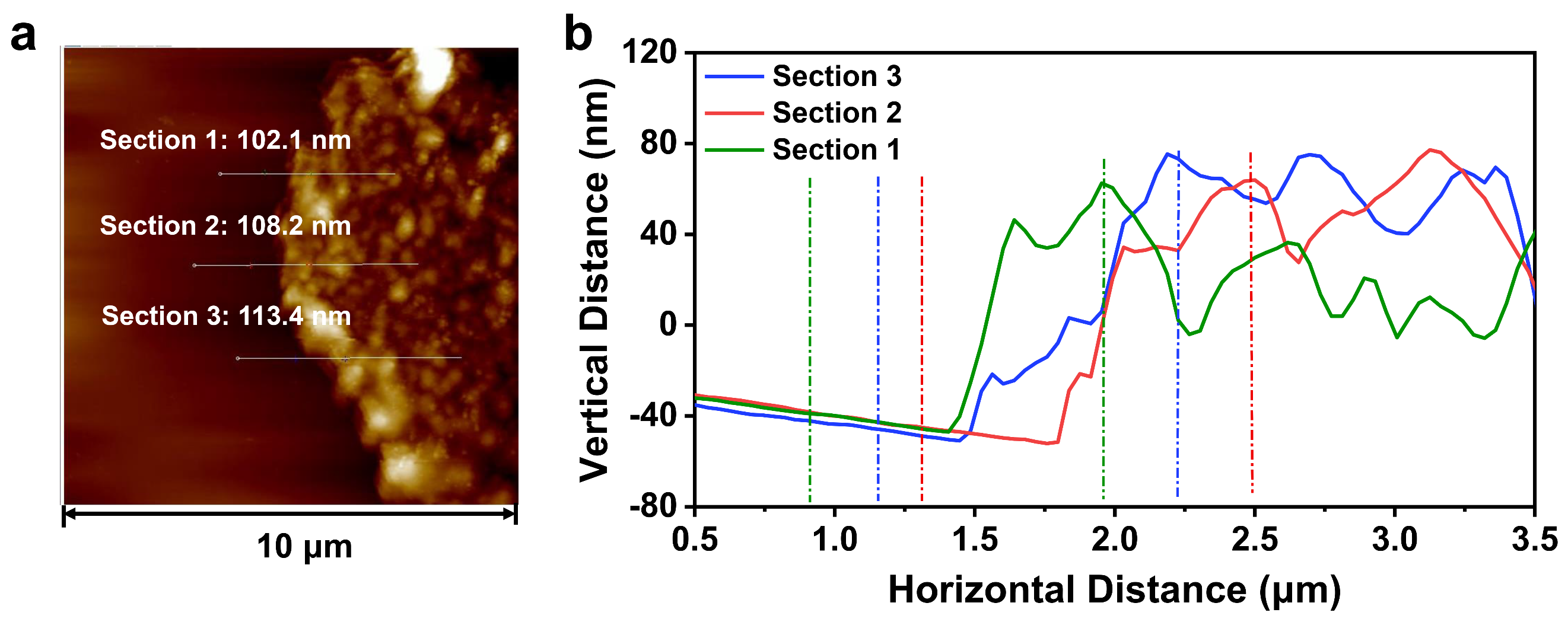


**Figure S3.** (a) AFM image of the PCOF membrane on tapping mode. (b) corresponding thickness analysis.

**Note:** The PCOF membrane displayed the thickness of 108 ± 6 nm by three different sections with the AFM analysis.


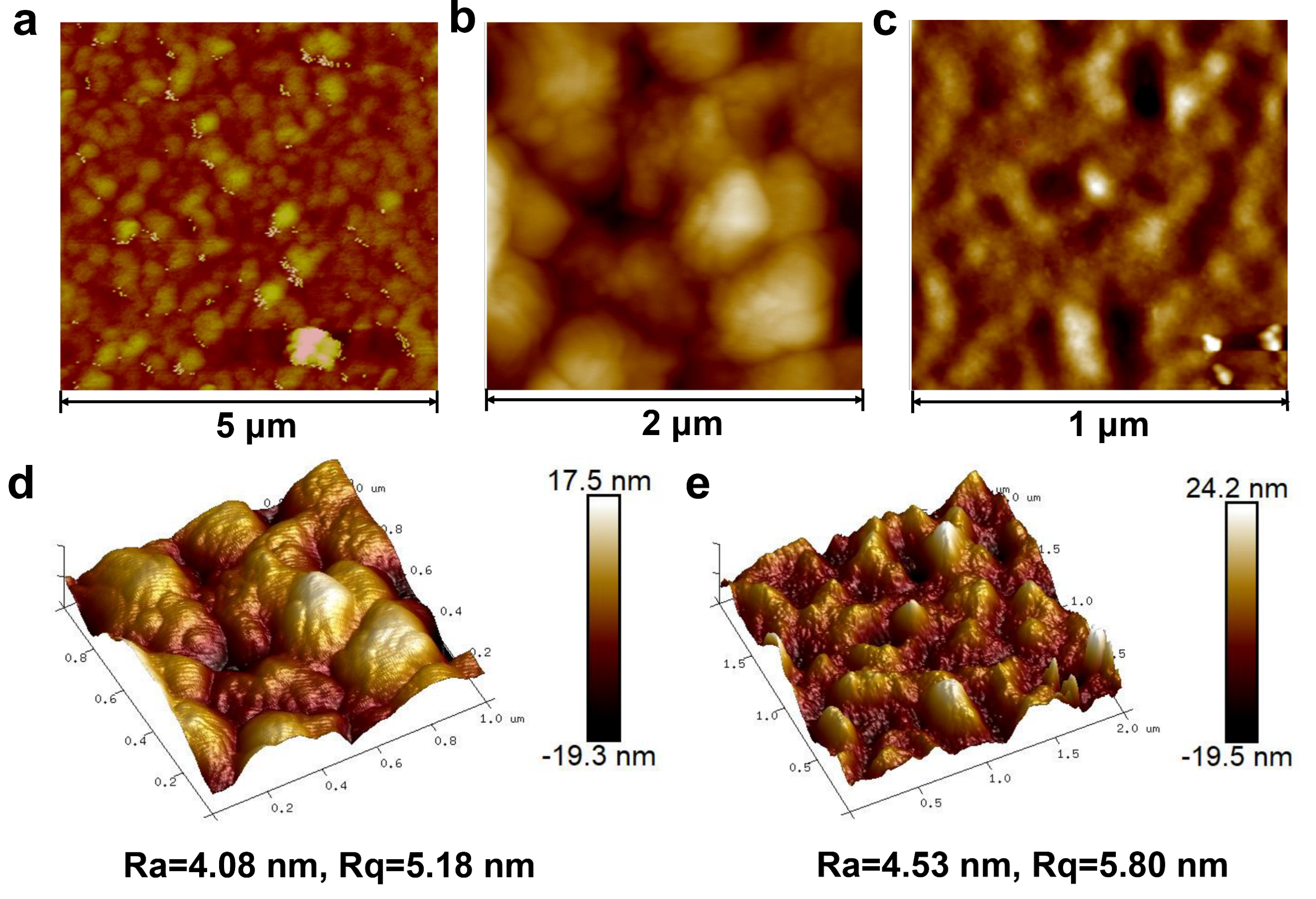


**Figure S4.** (a-c) AFM images of the PCOF membrane with different magnifications, (d) and (e) surface roughness of the PCOF membrane.

**Note:** The surface morphology was further characterized by AFM under different magnifications. The morphology indicated that structure of the PCOF membrane is the orderly lamellar arrangement of ionic domains similar to the cast Nafion (a kind of sulfonic-acid ionomer).^[1]^ The surface roughness of the PCOF membrane was evaluated by Ra and Rq. The results showed the PCOF membrane has quite smooth surface which is beneficial to resist salt crystallization.


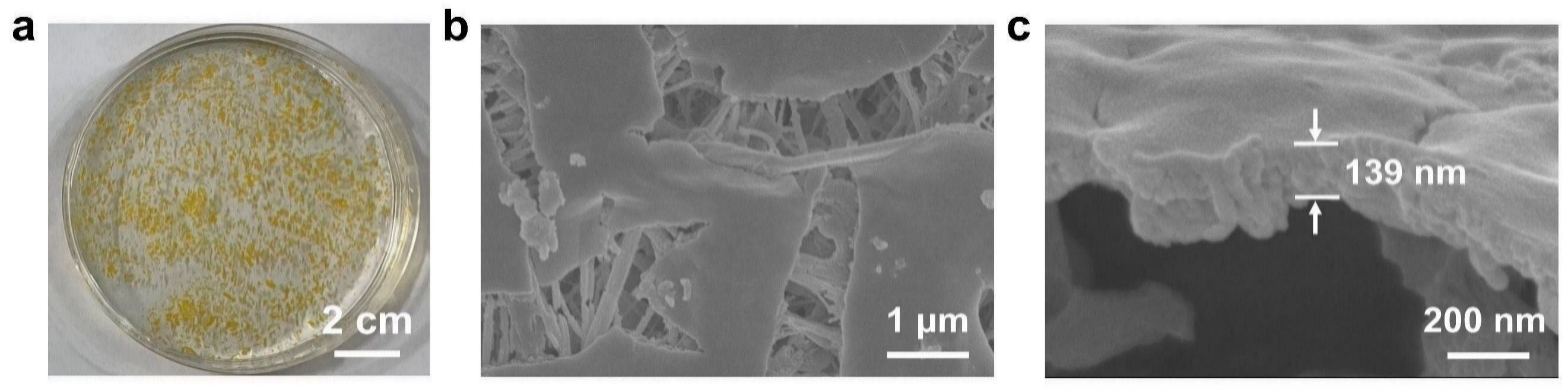


**Figure S5.** (a) SCOF membrane floating on the surface of water. (b) Surface SEM image of the SCOF membrane. (c) Cross-sectional SEM image of the SCOF membrane.

**Note:** The SCOF membrane prepared from liquid-liquid IP was transferred to the surface of water on which we can observe the membrane was spilt into small fragments because the molecular Vander Waals’ force and the π-π interaction between SCOF nanosheets are not enough to resist the surface tension of water. After deposited the porous polymer substrate, the SCOF membrane displayed large crack and the thickness of the SCOF membrane was 139 nm higher than the PCOF membrane.


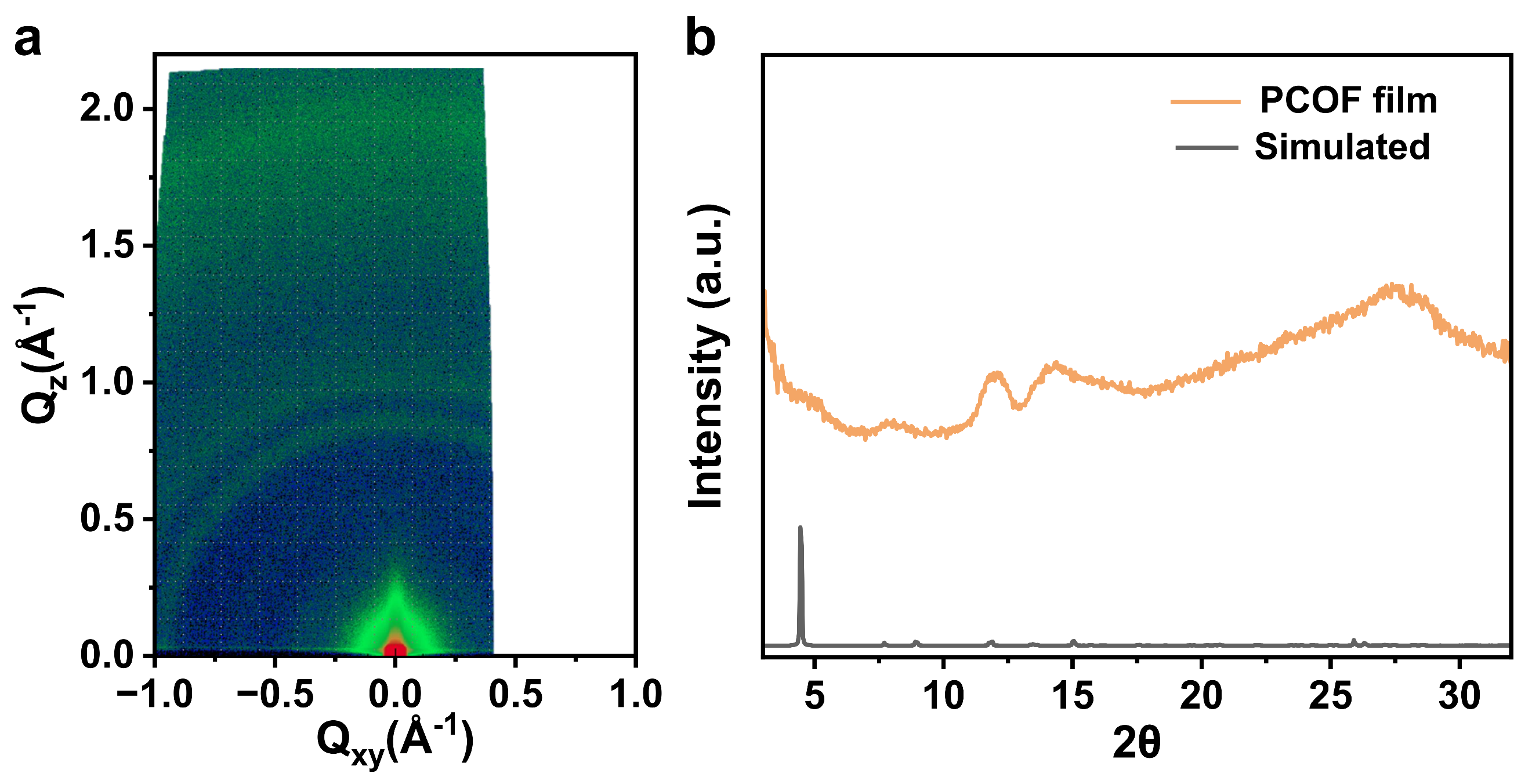


**Figure S6.** Grazing incidence small angle X-ray scattering (SAXS) characterization of the PCOF membrane.

**Note:** The SAXS characterization was applied to analyze the crystallinity of the PCOF membrane. The PCOF membrane at the oil-water interface was deposited on silicon wafer as the sample to be tested. The diffraction peak of (100) crystal plane was not strong enough mainly because the introduction of PDDA molecular chains will inevitably influence the spatially regular stacking of SCOF frameworks^[2]^. The (001) crystal plane located at 27.6° verified the lamellar structures.


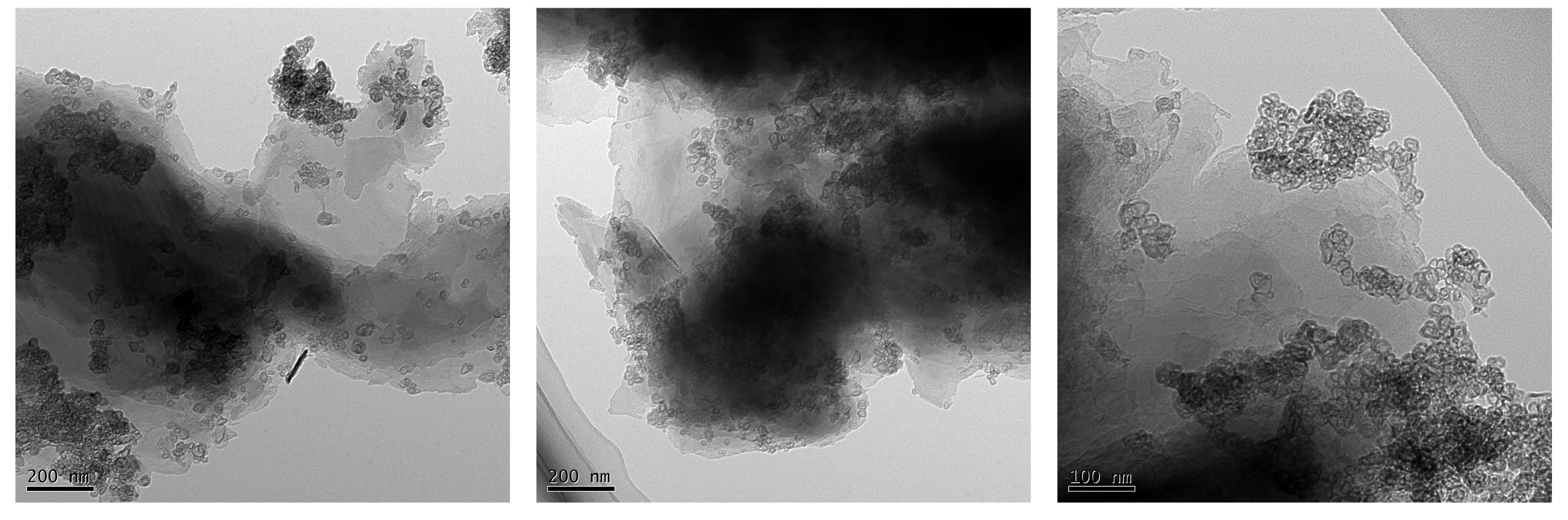


**Figure S7.** TEM images of PCOF membrane after ethanol ultrasonic treatment.

**Note:** Through ethanol ultrasonic treatment, the heterogenous structure of PDDA and SCOF nanosheets was destroyed and PDDA molecular chains present in the interlayer occurred crimp and separation due to the insolubility of PDDA in ethanol. Thus, SCOF nanosheets were stripped into thin layers. According to TEM images of different scales, PDDA molecular chains were mainly distributed at the edges of nanosheets by electrostatic attraction.


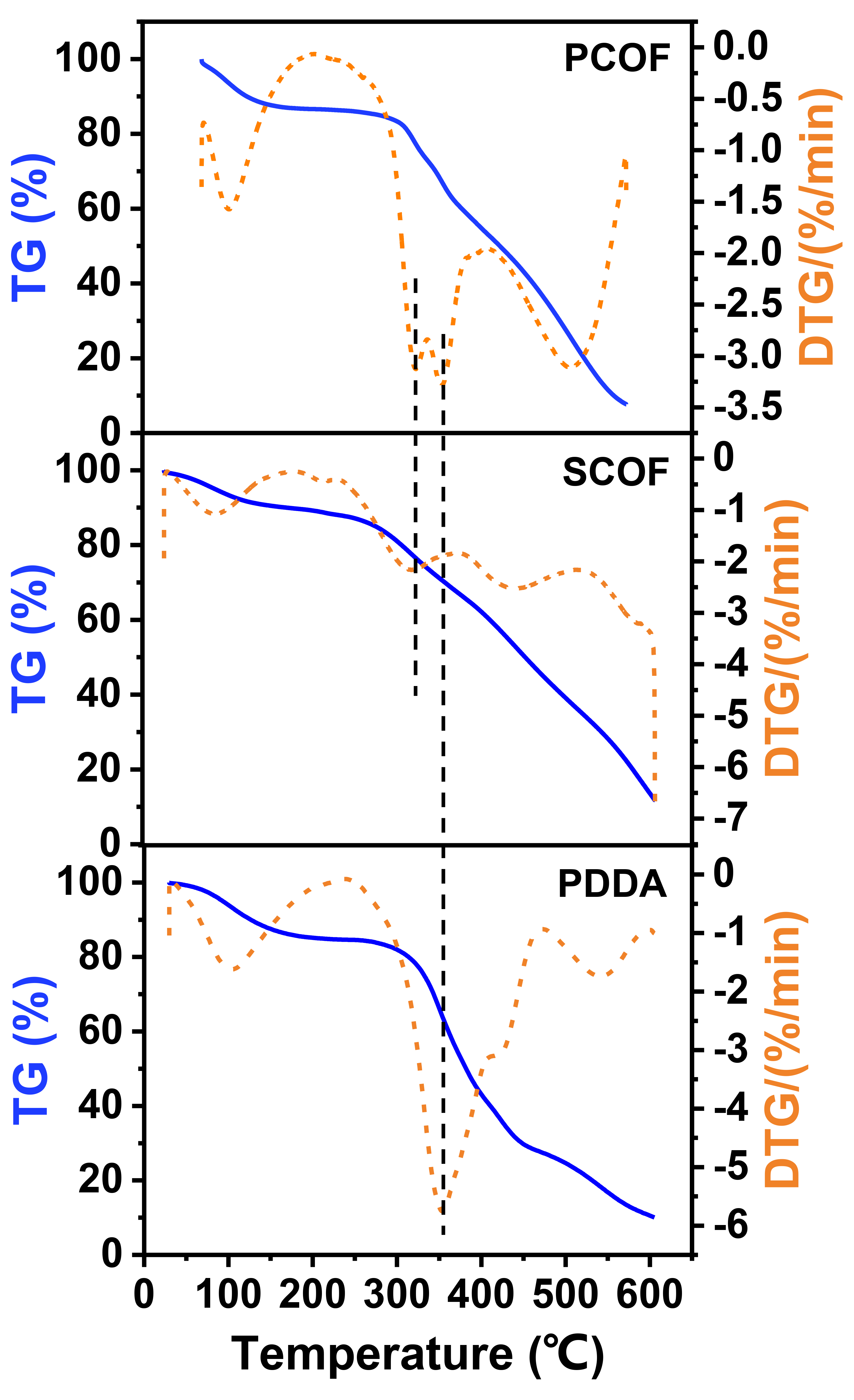


**Figure S8.** TG and DTG analysis of PCOF, SCOF and PDDA components.

**Note:** From the TG images, we can conclude that the PCOF component appeared two peaks at 300-400 ℃. The first peak position was 320.7 ℃ and the second peak position was 356.0 ℃, respectively corresponding to the ranges of thermal decomposition peaks of two components of SCOF and PDDA.


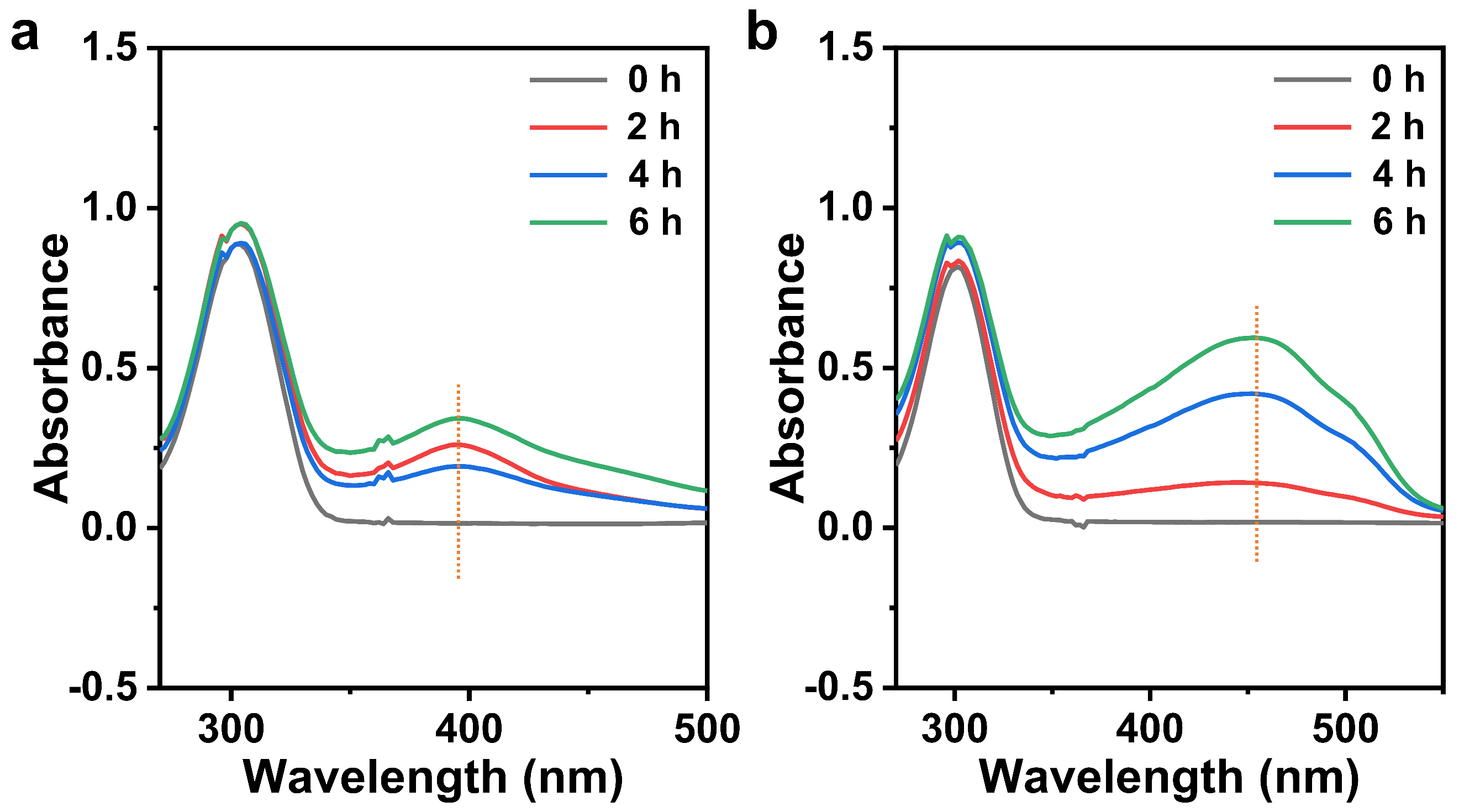


**Figure S9.** UV-vis spectra of SCOF components in the aqueous phases during IP reaction processes of (a) the SCOF membrane and (b) the PCOF membrane.

**Note:** The SCOF aqueous phase without PDDA and the PCOF aqueous phase with PDDA at different reaction time were conducted by UV-vis spectrum characterization. The specific peak of the amine monomer in the lower aqueous phase appeared at 304 nm which discloses the specific peak of the amine monomer. With the beginning of Schiff base reaction, the SCOF aqueous phase appeared a new peak at 396 nm, indicating the formation of SCOF conjugated structure. Besides, in the Figure S9a, the intensity of absorbance increasingly enhanced to 0.343 as the reaction time prolongs. Compared with the SCOF aqueous phase, the absorption peak of PCOF aqueous phase emerged at 454 nm with a red shift and more obvious increase in intensity, which implied the construction of larger conjugated system and more SCOF components at the same time (Figure S9b).


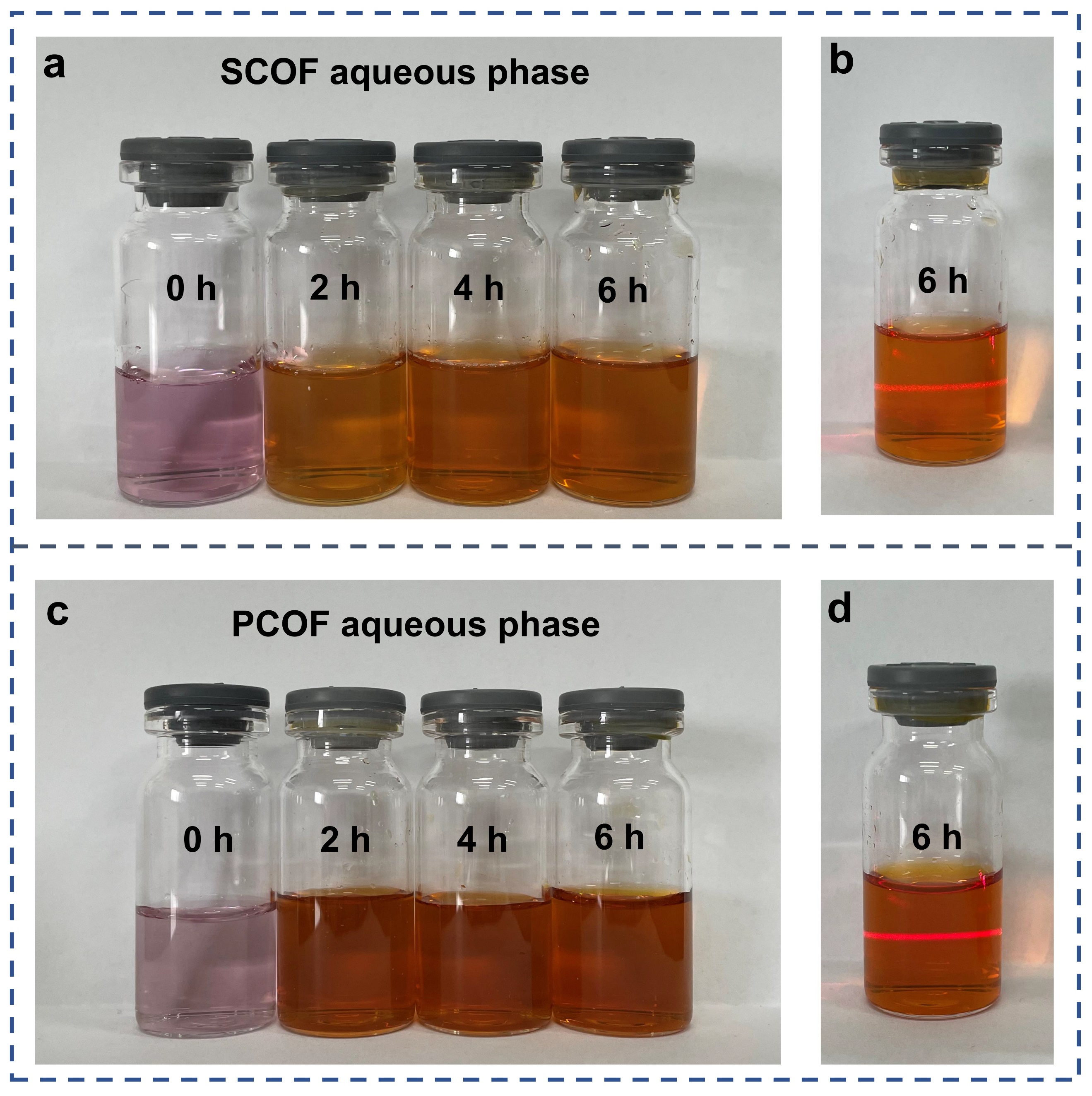


**Figure S10.** Optical images of different stages of SCOF aqueous phase or PCOF aqueous phase. (a) Color changes of SCOF aqueous phase with IP time from 0h to 6h. (b) Tyndall effect of SCOF aqueous phase at 6h. (c) Color changes of PCOF aqueous phase with IP time from 0h to 6h. (d) Tyndall effect of PCOF aqueous phase at 6h.

**Note:** We observed the color of aqueous phase increasingly deepened with the prolongation of IP time, which verified the diffusion direction of aldehyde monomers and the synthesis of SCOF units present in water phase. Compared with the reaction system of SCOF, the colors of aqueous phase of PCOF system got darker, implying the presence of PDDA accelerated the reaction process. Moreover, the Tyndall effect was more obvious in PCOF aqueous phase with IP time at 6h.


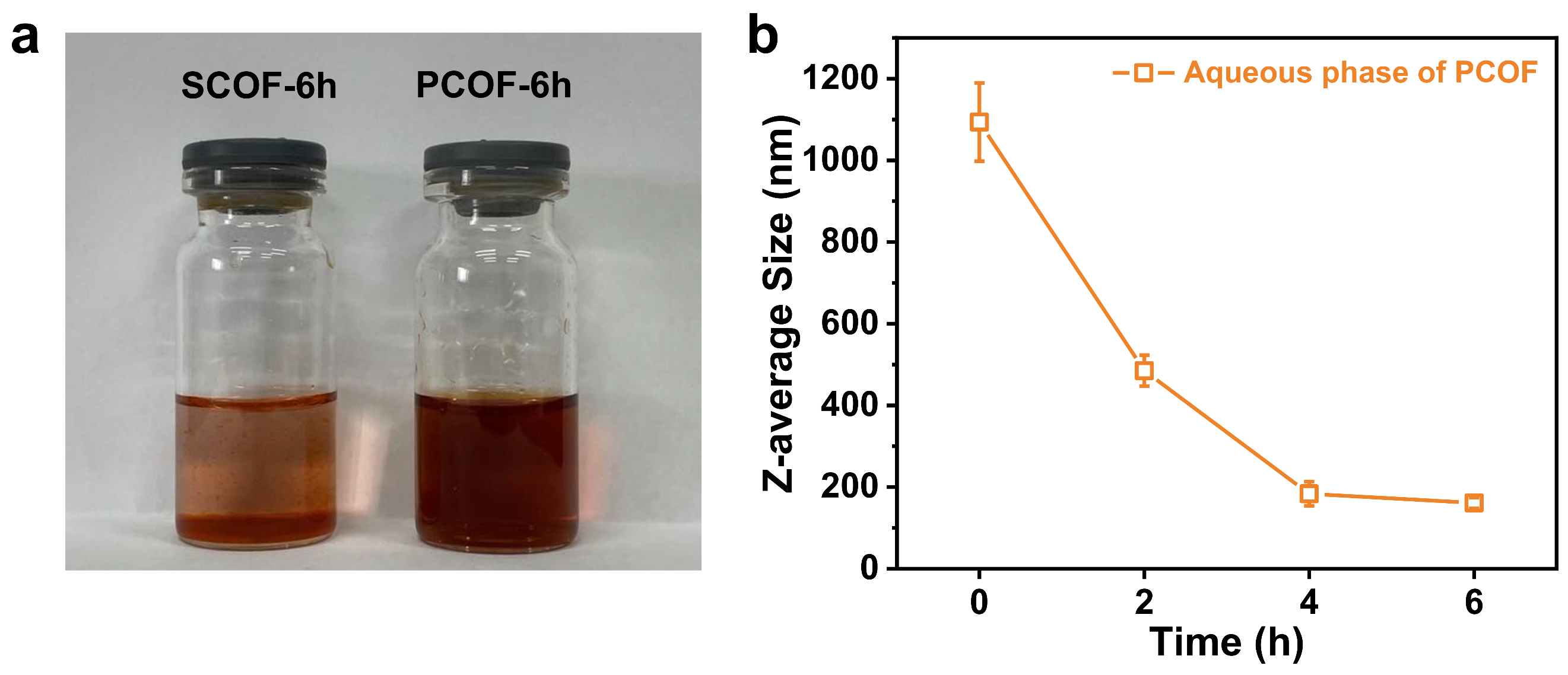


**Figure S11.** (a) Optical images of SCOF aqueous phase and PCOF aqueous phase with IP time at 6h after static settlement a certain time. (b) Variation of the hydrodynamic size in aqueous phase during IP reaction process of PCOF.

**Note:** After enough sedimentation by the self-weight of SCOF nanosheets, SCOF aqueous phase at 6h appeared obvious stratification, but PCOF aqueous phase showed ideal and homogenous status, indicating that PDDA with extremely strong hydrophilicity could stabilize the colloidal dispersion of SCOF nanosheets.


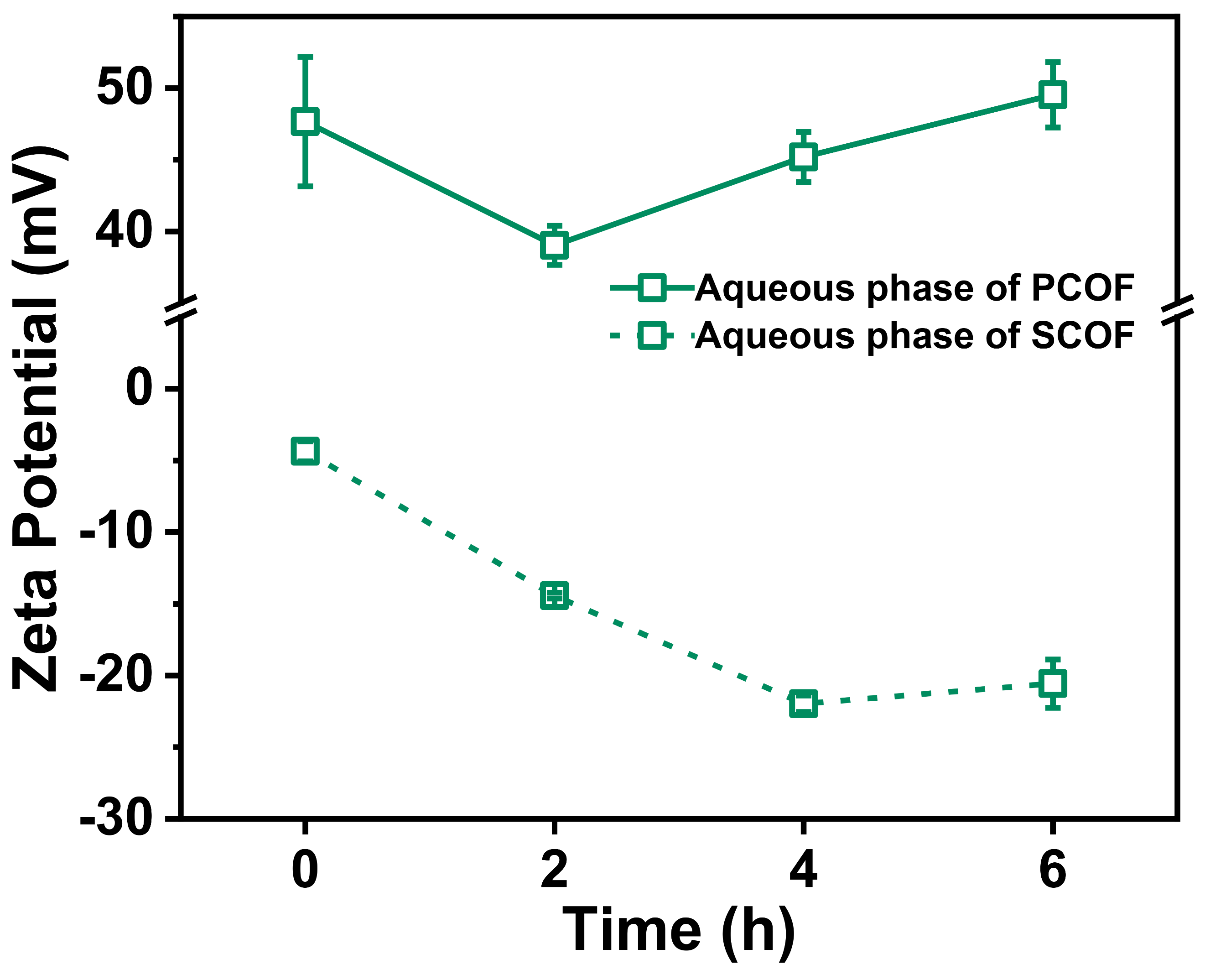


**Figure S12.** Variations of zeta potential in aqueous phase during the IP reaction process of the SCOF and PCOF membranes.

**Note:** The zeta potentials of PCOF and SCOF aqueous phases were measured with the IP reaction time increasing from 0 h to 6 h, the zeta potential of SCOF aqueous phase initially showcased negative value and gradually declines, finally tending to be stable, which could be attributed to the consumption of anime monomers. Comparatively, the potential of PCOF aqueous phase decreased within 2 h and gradually increased to the initially strongly positive potential, mainly because the presence of PDDA long chains shields the negative charges of SCOF by electrostatic and complex interactions.


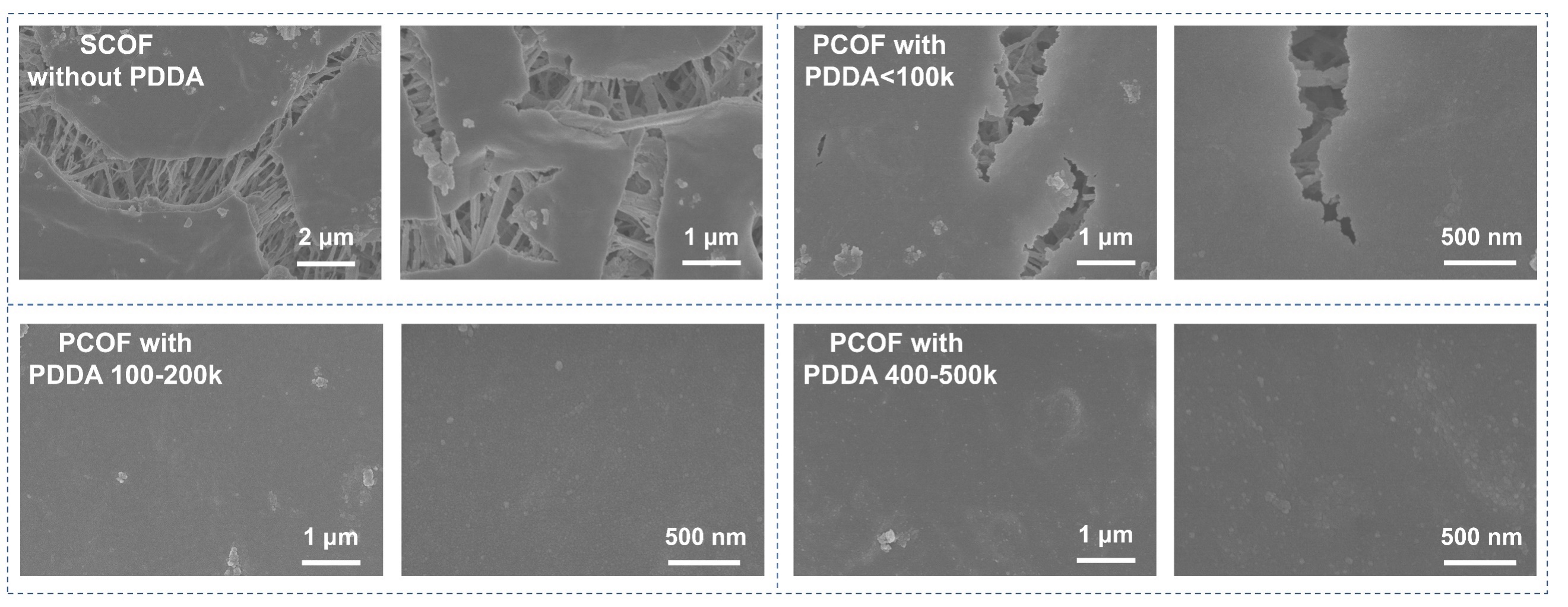


**Figure S13.** Surface SEM images of SCOF without PDDA, PCOF with PDDA<100k, PCOF with PDDA 100-200k, and PCOF with PDDA 400-500k membranes under different magnifications.

**Note:** the surface morphologies showed that the SCOF membrane was fragile and the PCOF with PDDA<100k membrane exhibited comparatively fewer cracks than SCOF membrane. The surfaces of the PCOF with PDDA 100-200k and PCOF with PDDA 400-500k membranes were totally defect-free and compact. Therefore, the integrity of the membrane is improved with the increasing molecular weight of PDDA.


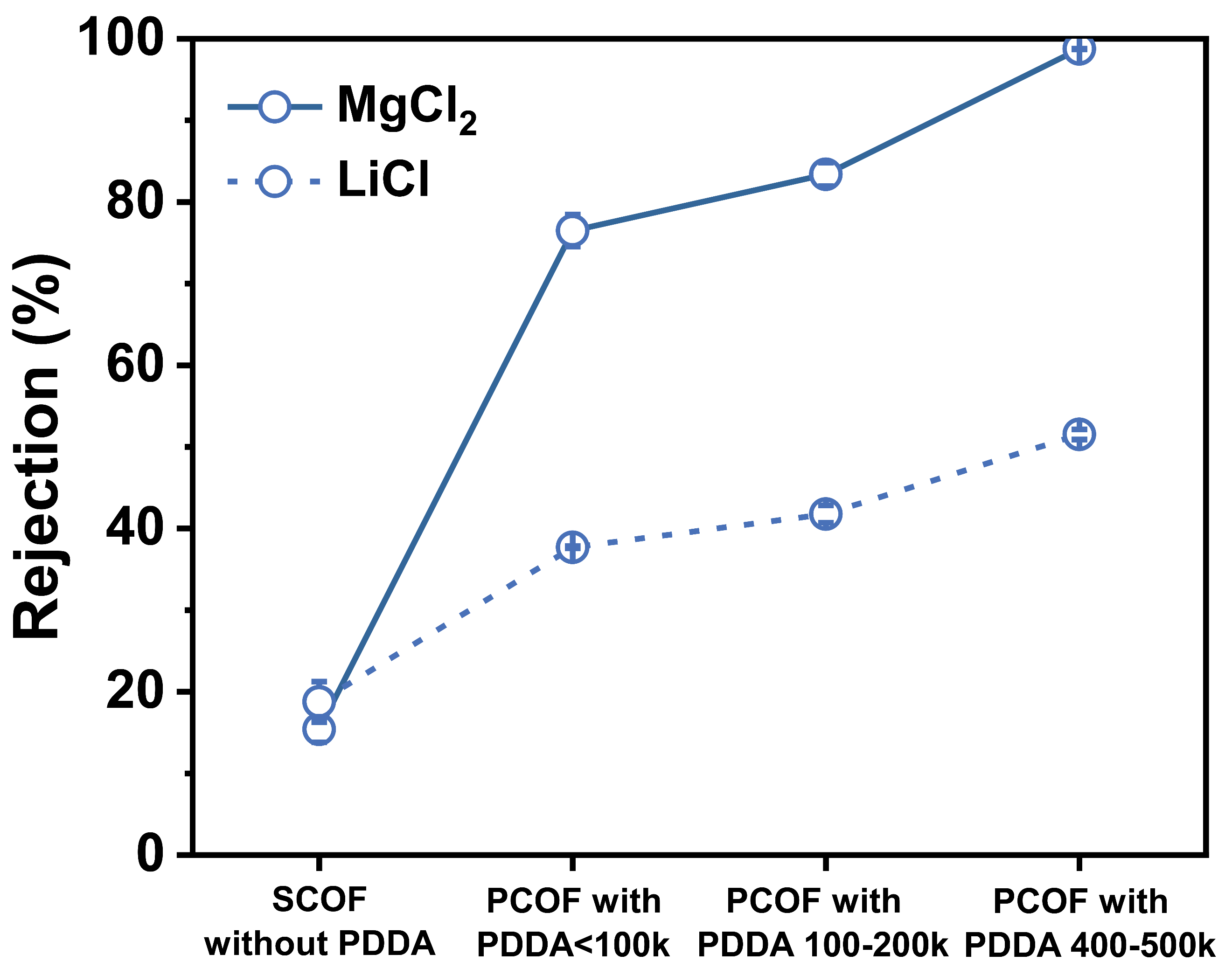


**Figure S14.** Rejection of MgCl_2_ and LiCl solutions of the SCOF without PDDA, PCOF with PDDA<100k, PCOF with PDDA 100-200k, and PCOF with PDDA 400-500k membranes.

**Note:** We selected a series of PDDA with diverse molecular weights to proceed IP process. The PCOF membranes were deposited on the polytetrafluoroethylene (PTFE) substrates as the PCOF thin membrane composite membranes to conduct desalination tests. The rejections of MgCl_2_ and LiCl solutions were utilized to evaluate the desalination performance. With the increasing molecular weight, the rejection of MgCl_2_ solution enhanced up to 98.8% used by PCOF with PDDA 400-500k membrane. The LiCl/MgCl_2_ selectivity of the PCOF with PDDA 400-500k membrane almost rose to 10 times compared with the membrane of PCOF with PDDA<100k, which elucidated that PDDA with sufficiently long chain and charged density is of vital significance to fabricate ion separation membrane. The concentration of feed solution is 1000 ppm.


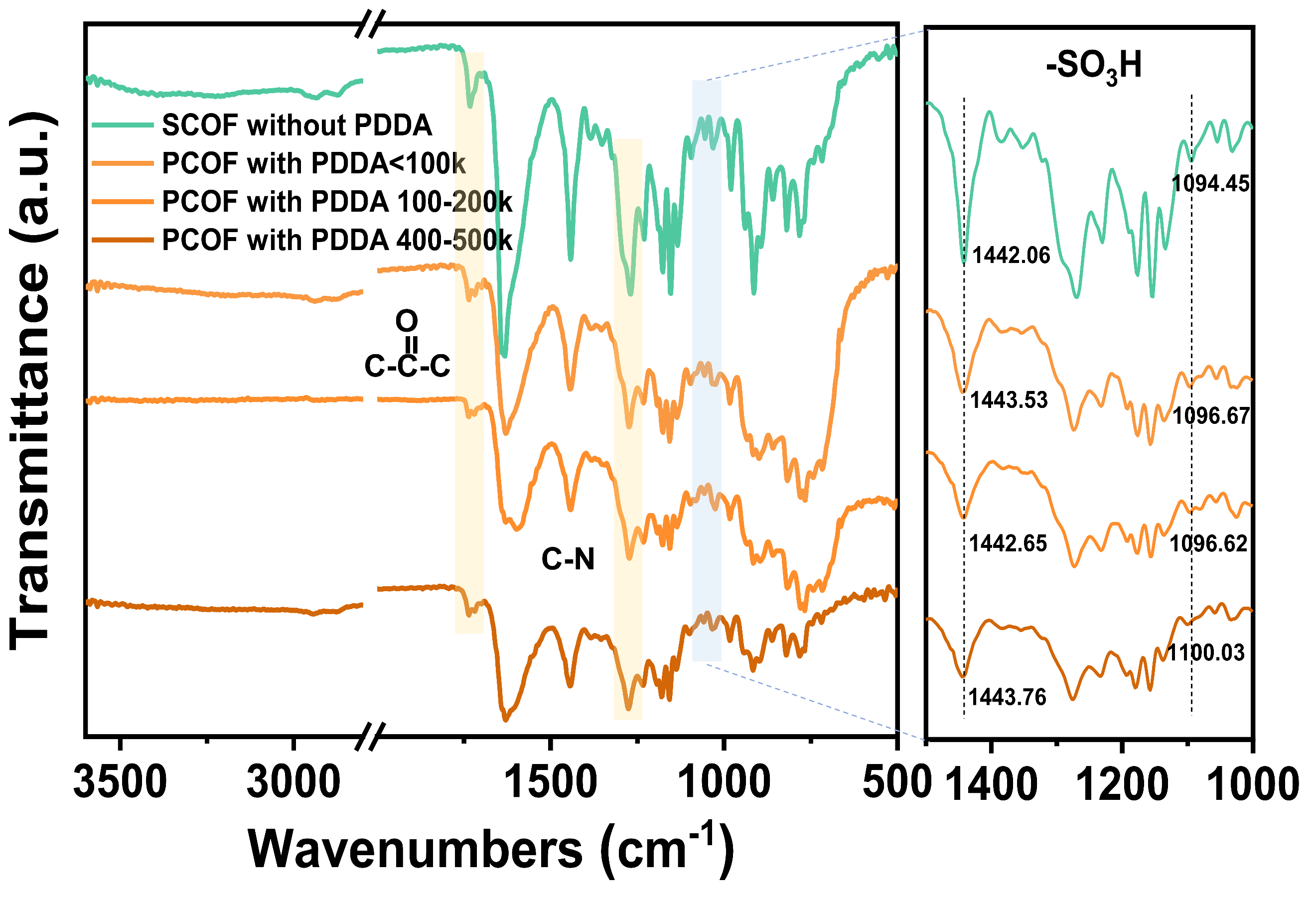


**Figure S15.** Microscopic infrared spectra of the SCOF without PDDA, PCOF with PDDA<100k, PCOF with PDDA 100-200k, and PCOF with PDDA 400-500k membranes, along with the variation of characteristic peak of sulfonic acid groups.

**Note:** The Micro-FTIR characterization of the SCOF membrane and the PCOF membranes with different molecular weights of PDDA firstly demonstrated the successful synthesis of TpPa-SO_3_H framework in view of the appearance of characteristic peaks of C-N and C=O. Compared with the SCOF membrane, the characteristic peaks of sulfonic acid group occurred red shift in the PCOF membranes, indicating the change of conjugated structure due to the electrostatic interaction between PDDA molecular chain and TpPa-SO_3_H components. The shift extent in the PCOF with PDDA 400-500k membrane was the largest than PCOF with PDDA<100k and PCOF with PDDA 100-200k, which verified that the molecular chain of PDDA is conducive to strengthening the electrostatic interaction.


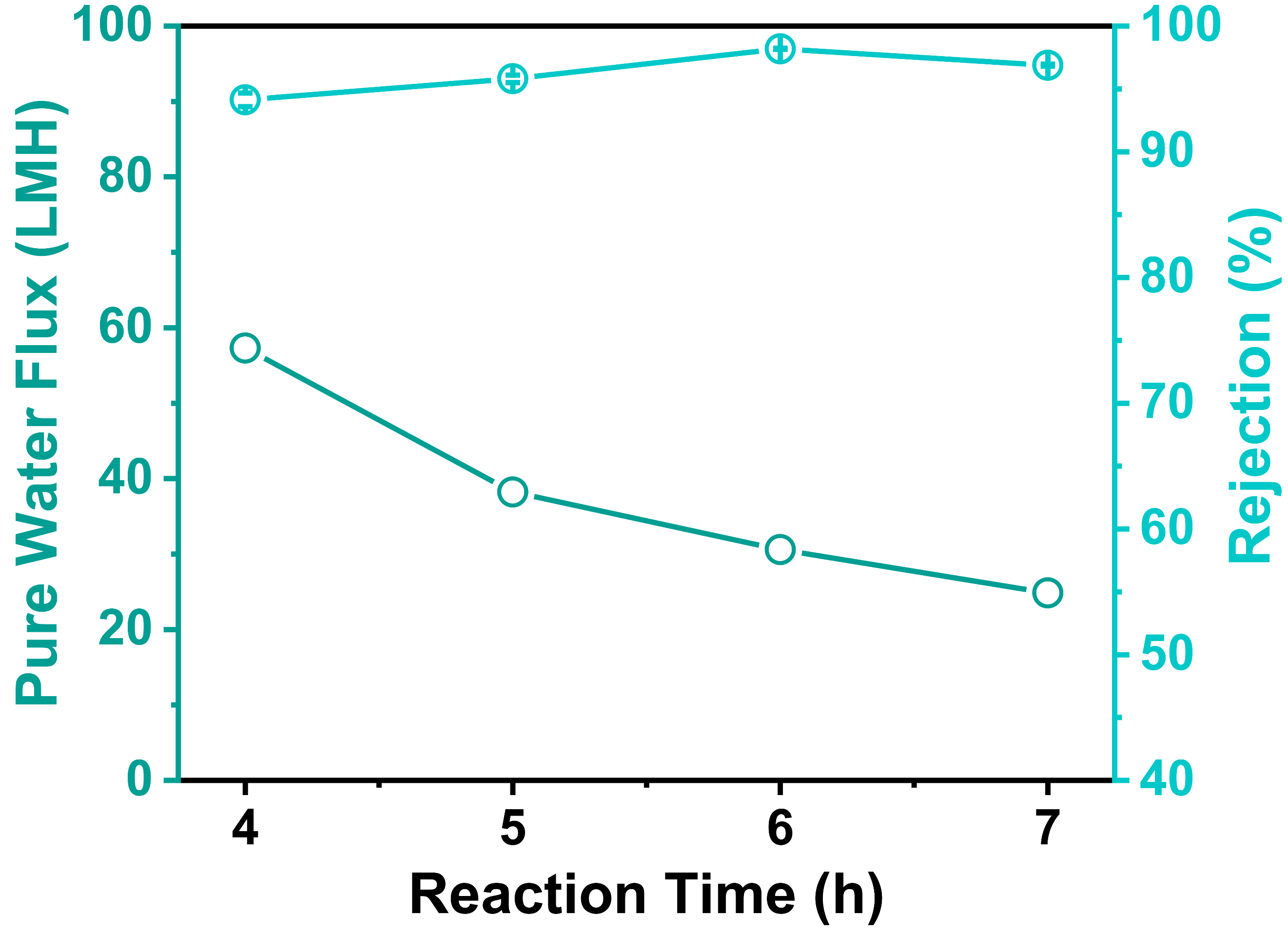


**Figure S16.** Separation performance of the PCOF membrane with 1000 ppm MgCl_2_ solution as the feed solution under different reaction time.

**Note:** We further adjusted the reaction time from 4 hours to 7 hours to determine the optimal reaction condition. The water flux gradually descended due to the increasing of reaction time. When the reaction time was 6 hours, the PCOF membrane performed the optimal rejection of MgCl_2_ solution in view of the match between the thickness and structure during the reaction process of IP. Compared with traditional methods to acquire self-standing COF membranes, the reaction time of polyelectrolyte-assisted strategy is greatly shortened.


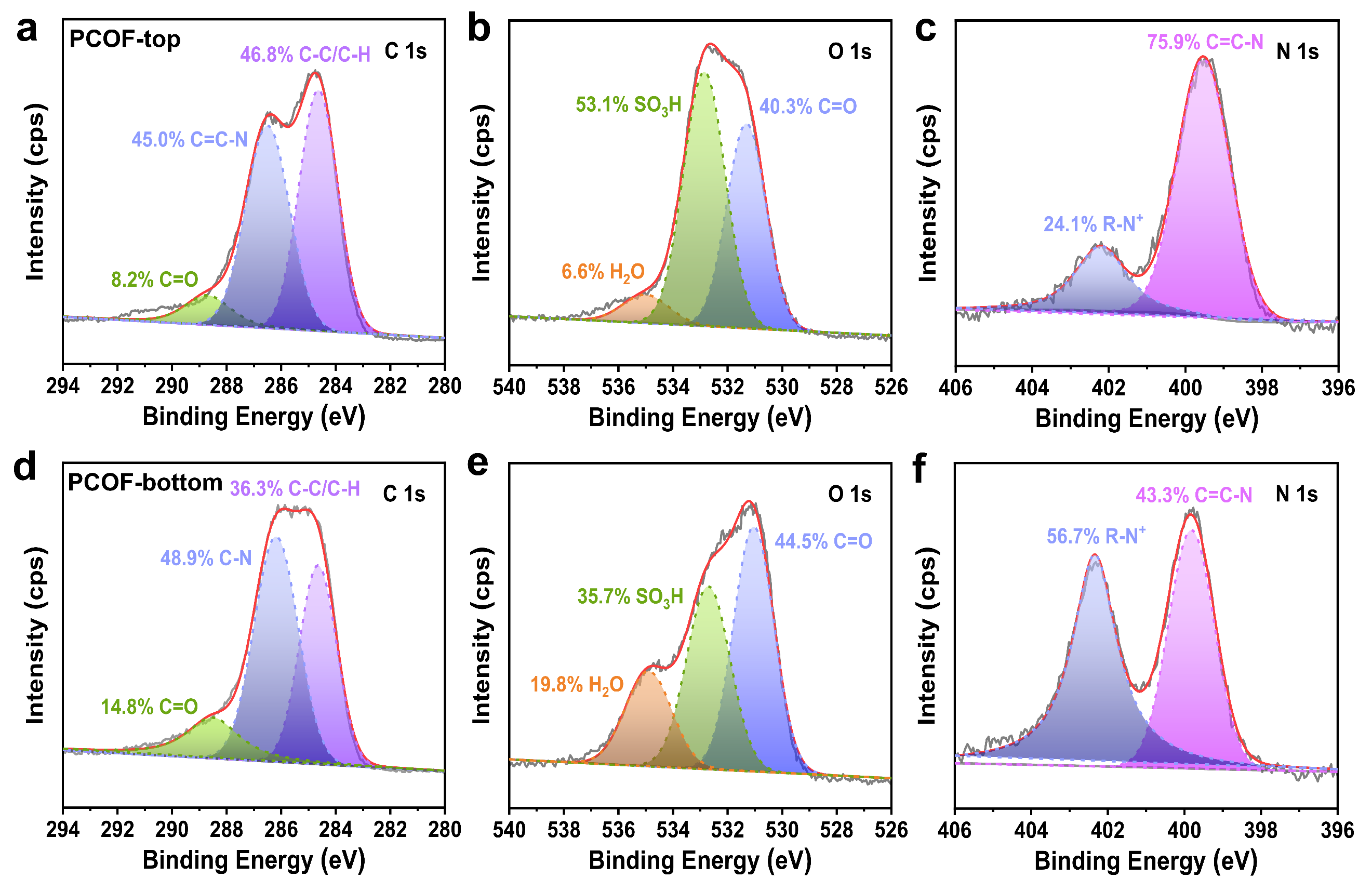


**Figure S17.** XPS analysis of the top of PCOF membrane with a) C 1s, b) O 1s, and c) N 1s. XPS analysis of the bottom of PCOF membrane with d) C 1s, e) O 1s, and f) N 1s.

Note: The XPS characterizations of both sides of the PCOF membrane contained three primary spectra of C 1s, O 1s, and N 1s. The high-resolution C 1s deconvolution spectrum of the top of PCOF membrane showed three peaks that are associated with C-C/C-H at 284.6 eV, C=C-N at 286.2 eV, and C=O at 288.7 eV (Figure S17a). The O 1s spectrum displayed the characteristic peak for SO_3_H, which verified the presence of SCOF component. The peak at 535.0 eV was associated with the chemisorbed oxygen due to the hydrophilic surface (Figure S17b). The N 1s spectrum exhibited two typical peaks that C=C-N at 399.5 eV and R-N^+^ at 402.3 eV respectively represented the components of SCOF and PDDA (Figure S17c). The bottom of PCOF membrane had the similar peak-splitting situations, but the content of SO_3_H wad less and the content of R-N^+^ was more than the upper surface, elucidating that more PDDA was present in the bottom of the PCOF membrane (Figure S17d-f).


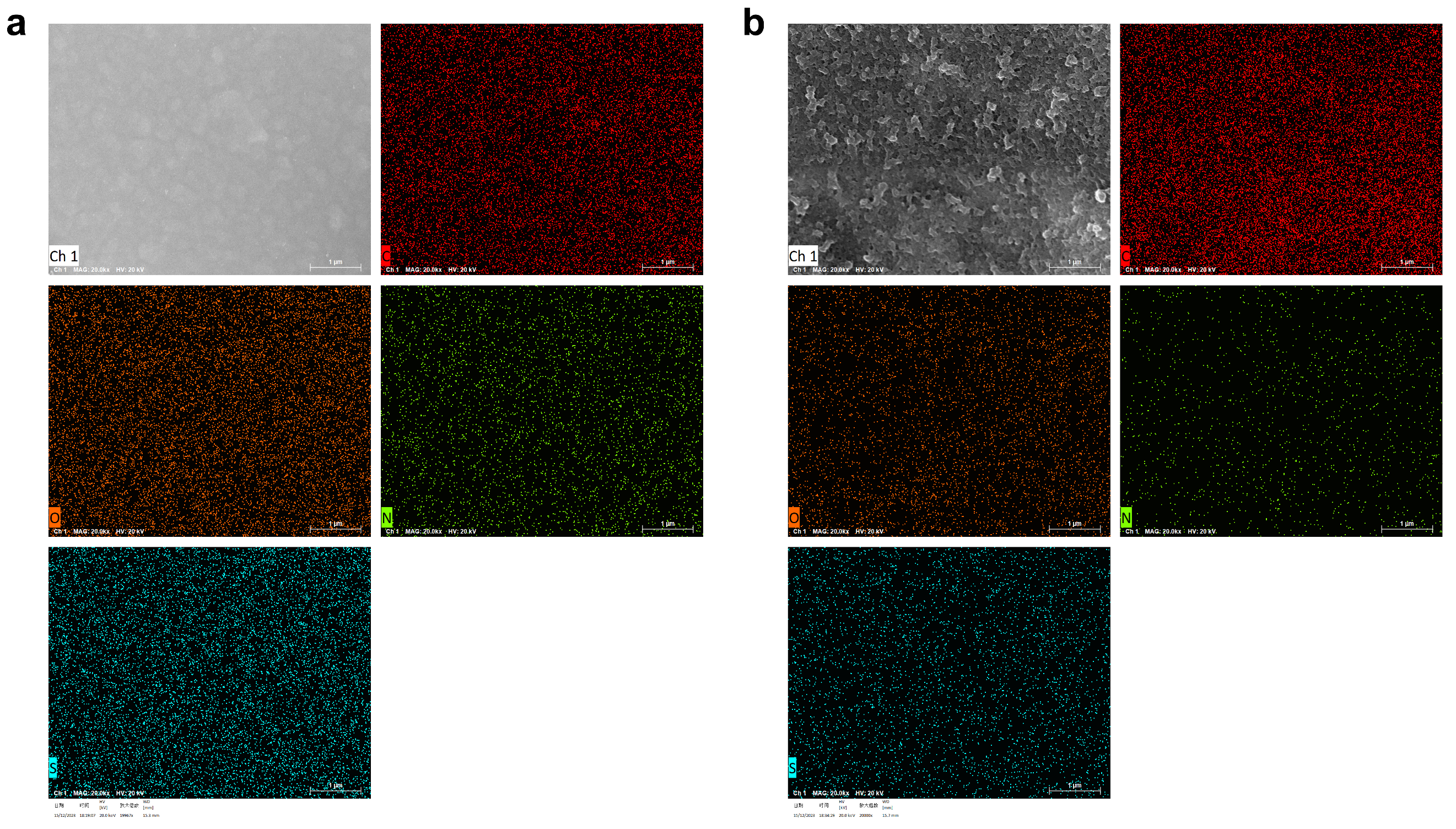


**Figure S18.** Elements distribution including C, O, N, and S of **a** top surface; **b** bottom surface of PCOF membrane by EDS mapping analysis.

**Note:** The elements of C, O, N, and S of both sides of the PCOF membrane were evenly distributed.


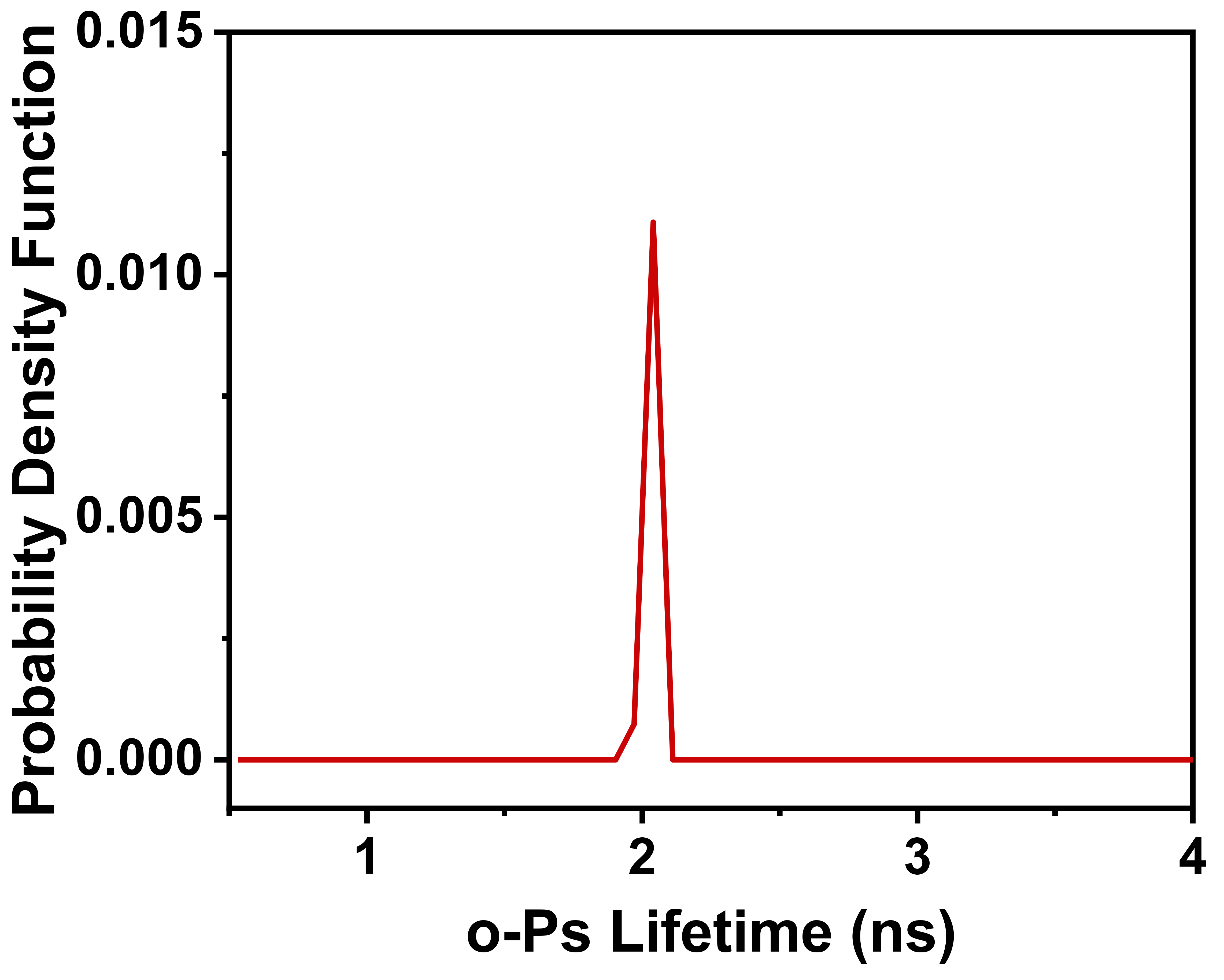


**Figure S19.** oPs result from PALS.

**Note:** PALS utilizes ^22^Na as the source of positrons. When positrons and electrons collide, they will respectively produce paraPositronium (pPs) and orthpositronium (oPs). PALS uses oPs with the longest life τ_3_ to determine the size and number of free volume elements in pre-tested materials. As the free volume gets larger, the oPs lifetime increases. Accordingly, the mean free volume radius (*R*) can be calculated by *τ_3_* using the following semi-empirical equation (1).^[3]^

$\tau_{3}=\frac{1}{2}[1-\frac{R}{R_{0}}+\frac{1}{2\pi}sin(\frac{2\pi R}{R_{0}})]$ (1)

Where *R* is the radius of the free-volume elements and *R_0_= R + ΔR* (where *ΔR* is 1.66 Å related to the electron thickness layer). The fractional free volume (*FFV*) is calculated by the following equation (2).

$FFV=C\frac{4}{3}\pi R^{3}I_{3}$ (2)

Here, *C* is an empirical constant determined to be 0.0018 nm^-3^, *R* is the mean free volume radius, and *I_3_* is the intensity of oPs.


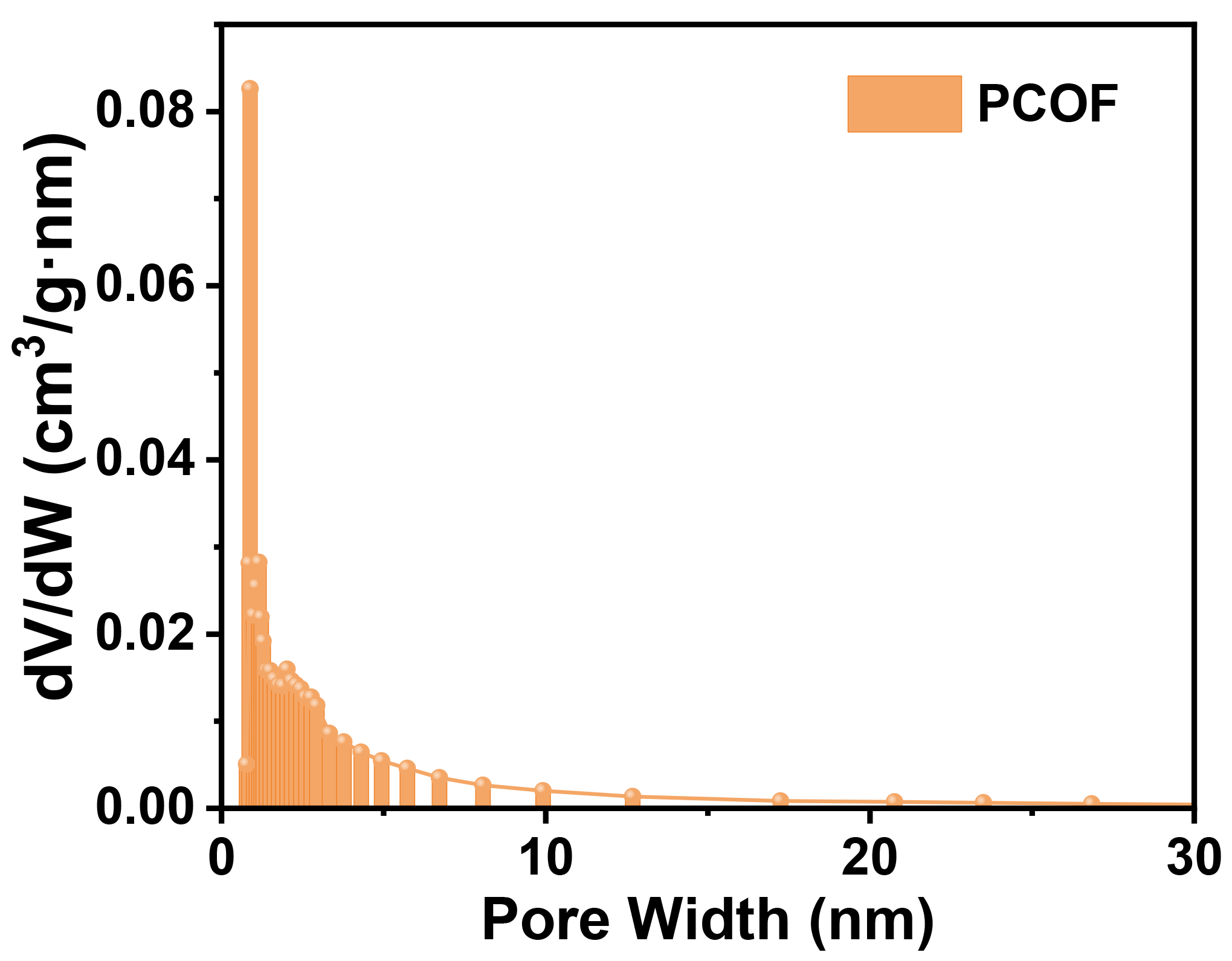


**Figure S20.** The pore size distribution of the PCOF flakes characterized by BET analysis.

**Note:** We conducted Brunauer-Emmett-Teller (BET) characterization utilized carbon dioxide as the adsorption gas to reflect the pore structure of PCOF nanoflakes. The PCOF nanoflakes were collected from aqueous phase and prepared into powder after freeze-drying treatment. The results showed that the micropore size of PCOF nanoflakes was 0.88 nm which was slightly bigger than the channel aperture (~0.576 nm) of PCOF membrane. It is thought that the assembly of nanosheets and the intercalation of PDDA chains confined at the interface caused a shielding effect and shrink the actual channel size.


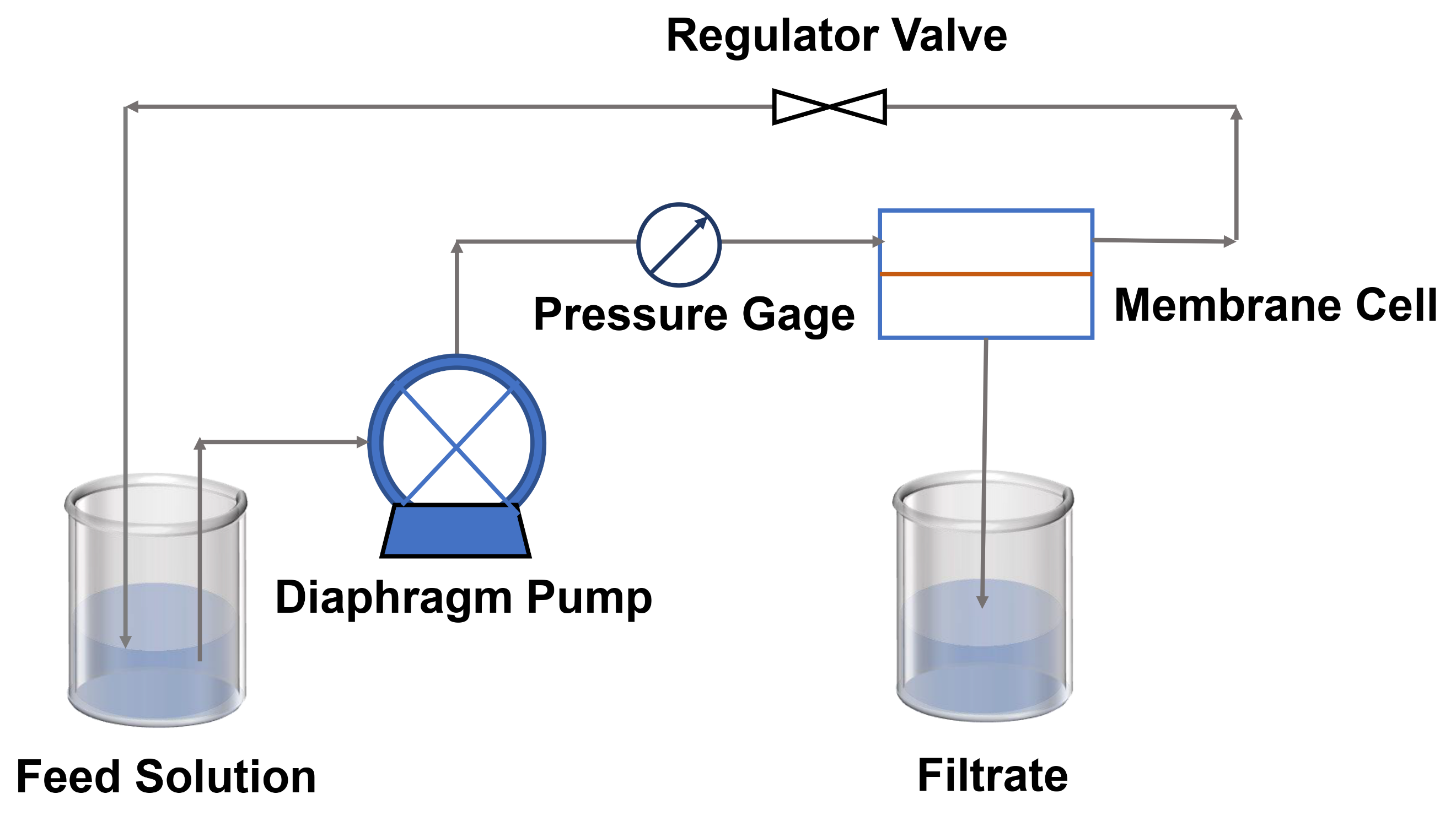


**Figure S21.** Schematic illustration of cross-flow filtration apparatus.

**Note:** All desalination and ion separation experiments were conducted by this cross-flow filtration apparatus. The PCOF membrane installed into the membrane cell was compacted with rubber ring and the effective filtration area was 3.14 cm^2^. In the membrane cell, one side was the circulating feed solution and the other side was the filtrate. The feed solution concentration of the mixed salts was 2000 ppm and the feed solution concentration of the single salt was 1000 ppm. The diaphragm pump can provide enough pressure to produce freshwater. The range of pressure gage was 1 to 10 bar. We can adjust the regulator valve to obtain desirable operational pressure.


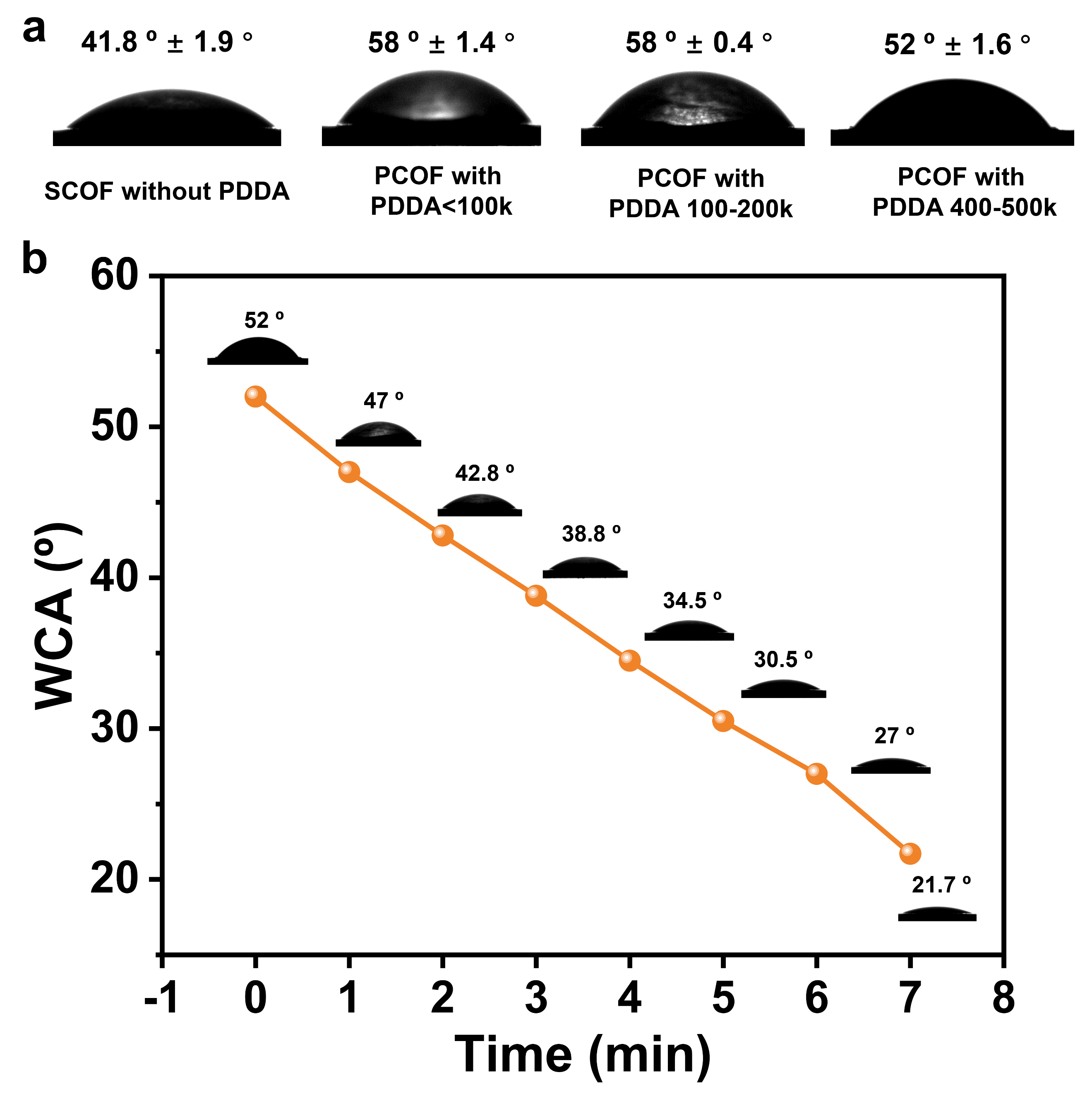


**Figure S22.** (a) WCA performance of SCOF without PDDA and PCOF with diverse molecular weights of PDDA. (b) Water wettability of the PCOF membrane.

**Note:** The SCOF without PDDA, PCOF with PDDA<100k, PCOF with PDDA 100-200k, and PCOF with PDDA 400-500k membranes showed excellent hydrophilicity that the water contact angles were lower than 90°. For the PCOF membrane, the water wettability is desirable according to the decreasing WCA within several minutes.


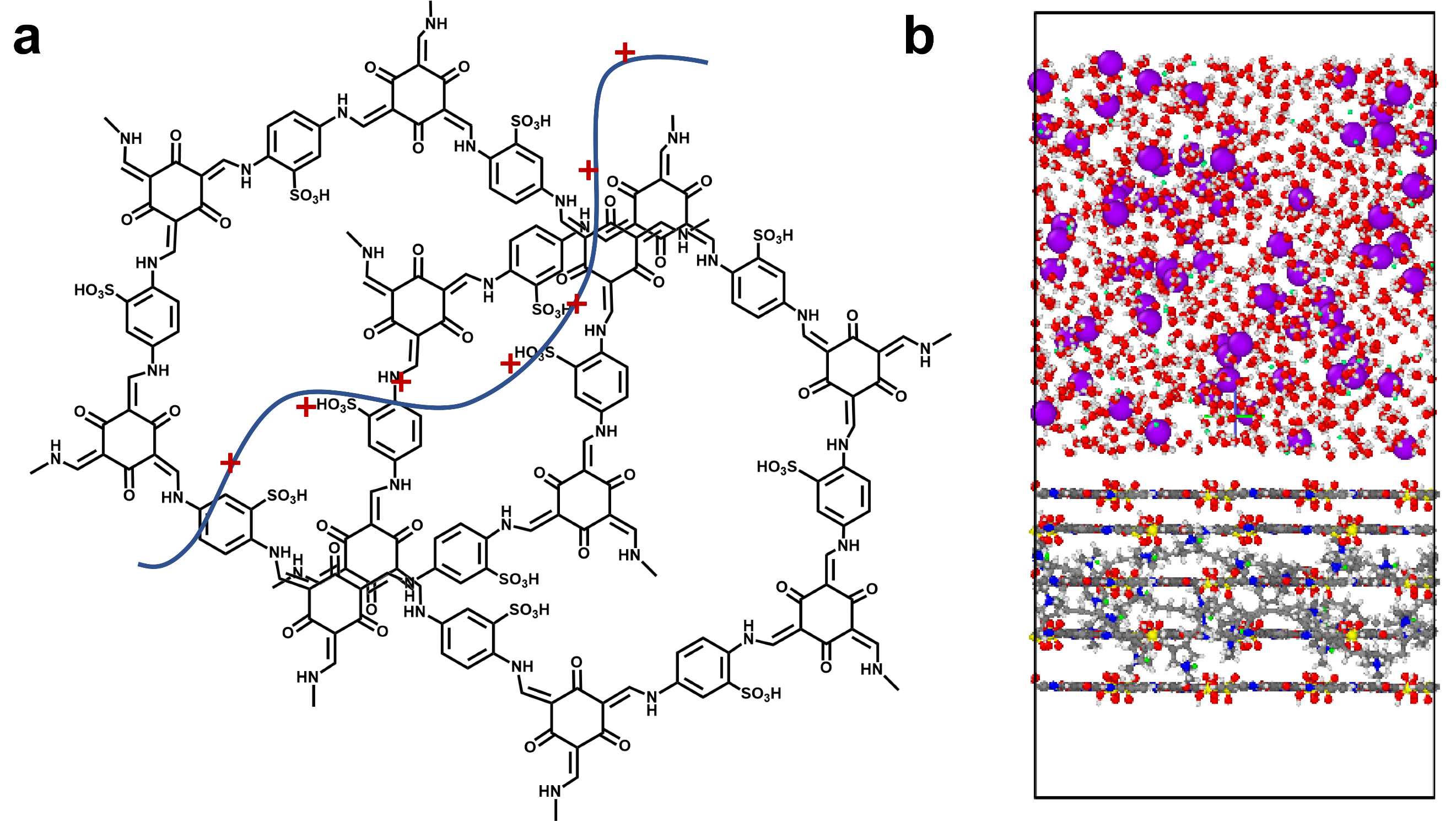


**Figure S23.** (a) Molecular structure of the PCOF membrane (mainly eclipsed stacking mode of SCOF nanosheets caused by the polycation). (b) Simulation box.

**Note:** Classic molecular dynamics (MD) simulations were performed to reveal the permeation behaviors of solution composed of MgCl_2_/LiCl and water through the PCOF membrane. The top-view of the molecular structure of PCOF membrane is shown in Figure S22a. In this study, the PCOF membrane was composed of five layers that were stacked in staggered fashion. As a result, as-prepared multilayered PCOF membrane showed unique pores that run parallel to the direction of stacking. Consideration of heterogeneous structure and charged gradient distribution of the ion channel, diverse PDDA polymer chains were inserted between the SCOF layers. Here, the second and third interlayered gaps were inserted by one and two PDDA molecules. The MD box had dimensions of 40 × 45 × 90 Å^3^, in which the top and middle parts were occupied by MgCl_2_ or LiCl solution and the PCOF membrane, respectively, whereas the bottom part was vacuum, as shown in Figure S22b. Periodic boundary conditions (PBCs) were imposed in the x- and y-directions, while the z-direction was wall boundary condition. To describe the atomic interactions in the PCOF membrane, consistent valence forcefield (CVFF)^[4]^ was utilized. The water molecule was described by the common simple point charge (SPC) model.^[5]^ The non-bonded interaction parameters of cations and anions were taken from Refs.^[6]^ For the non-bonded interactions in the system, a cutoff distance of 12 Å for both the standard Coulomb and 12-6 Lennard-Jones potentials was assigned to describe the van der Waals (vdW) forces and atomic electrostatic interactions.

Prior to MD simulations, energy minimization was firstly performed to relax the salt solution in the system. Then, MD simulations with 100 ps were performed to further relax the solution under canonical (NVT) ensemble at temperature of 300 K, in which the temperature was controlled by the Nose-Hoover thermostat. Finally, production simulations of nonequilibrium MD (NEMD) running with 10 ns at 300 K under NVT ensemble were performed to investigate the permeation behavior of cations through the PCOF membrane. Here, a pressure drop (ΔP) of around 50 MPa across the PCOF membrane was imposed by an external force that was achieved by exerting to ions and water molecules, which was expressed as $\text{ΔP=}\frac{\text{n}_{\text{ions}}\text{f+}\text{n}_{\text{water}}\text{f }}{\text{A}}$, where *n_ions_* and *n_water_* are the numbers of ions and water molecules, respectively, and *f* and *A* are the constant force exerted on species and the cross-sectional area of the PCOF membrane. Note that the constant force exerted on water molecule was only applied on oxygen of water. During the whole MD simulations, the PCOF membrane and PDDA chains were frozen. A timestep of 1.0 fs was used to integrate the Newton’s motion equation in the systems by the velocity-Verlet algorithm. All the MD simulations were implemented using the Large-scale Atomic/Molecular Massively Parallel Simulator (LAMMPS) package.^[7]^


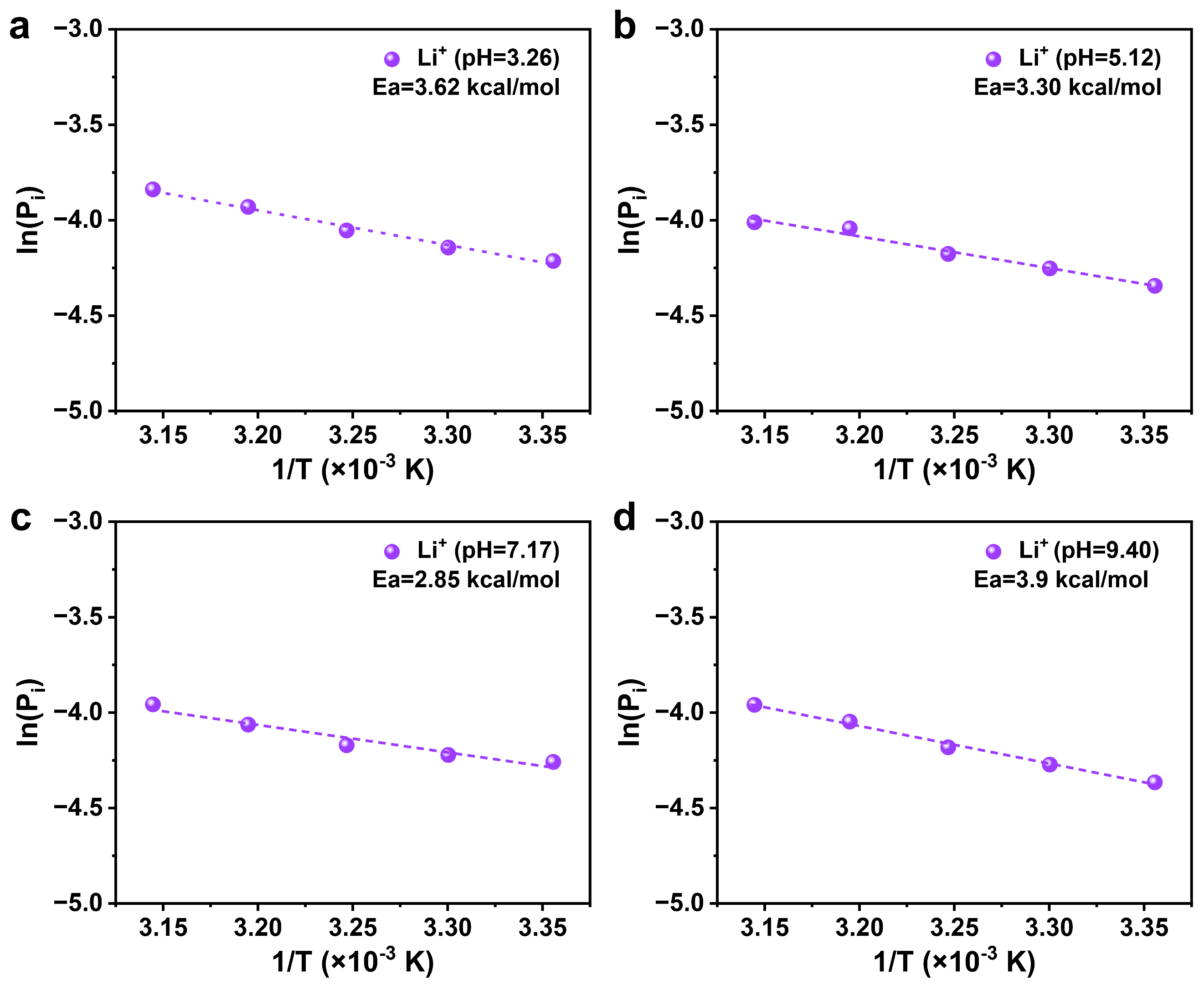


**Figure S24.** The linear relation between the natural log of permeation rate P_i_ of Li^+^ and the reciprocal of absolute temperature T under different pH conditions: (a) pH=3.26, (b) pH=5.12, (c) pH=7.17, and (d) pH=9.40.

**Note:** According to the Arrhenius-type equation, the osmosis diffusion cell was used to evaluate the transport energy barriers. The feed solution was the mixed salt solutions containing 0.1 M MgCl_2_ and 0.1 M LiCl. The Li^+^ concentration of permeation side was obtained by ICP-OES characterization. The slope of the linear fit was the energy barrier E_a_ divided by the gas constant R. The calculated E_a_ of Li^+^ at pH=3.26, 5.12, 7.17, and 9.40 were respectively 3.62, 3.30, 2.85, and 3.90 kcal/mol.


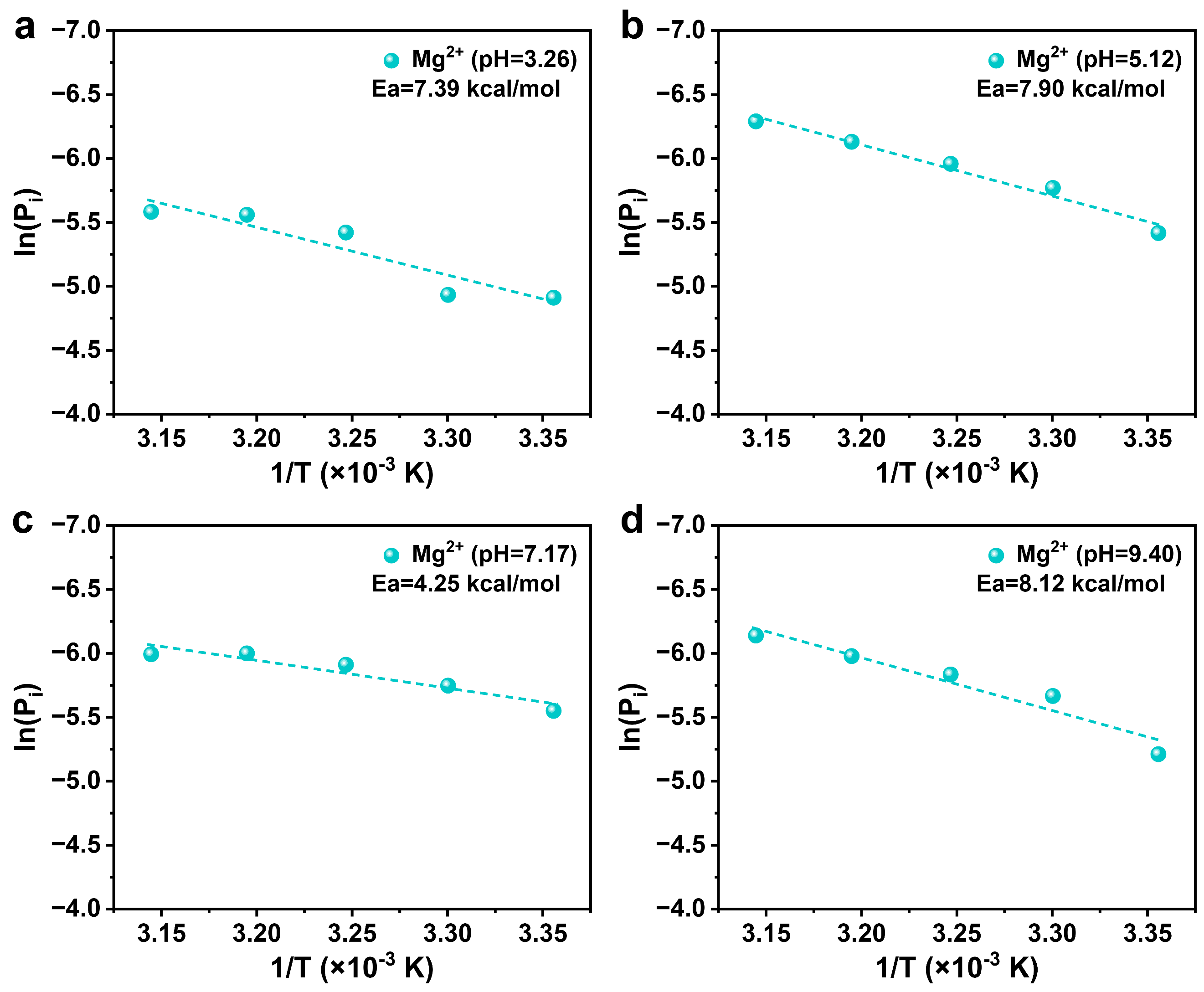


**Figure S25.** The linear relation between the natural log of permeation rate P_i_ of Mg^2+^ and the reciprocal of absolute temperature T under different pH conditions: (a) pH=3.26, (b) pH=5.12, (c) pH=7.17, and (d) pH=9.40.

**Note:** According to the Arrhenius-type equation, the osmosis diffusion cell was used to evaluate the transport energy barriers. The feed solution was the mixed salt solutions containing 0.1 M MgCl_2_ and 0.1 M LiCl. The Mg^2+^ concentration of permeation side was obtained by ICP-OES characterization. The slope of the linear fit was the energy barrier E_a_ divided by the gas constant R. The calculated E_a_ of Mg^2+^ at pH=3.26, 5.12, 7.17, and 9.40 were respectively 7.39, 7.90, 4.25, and 8.12 kcal/mol.

# Section S-7: Tables

**Table S1.** Mass fraction and atomic fraction of elements (C, O, N, and S) of both sides of the PCOF membrane by EDS mapping analysis.

| Element | Top | | Bottom | |
| --- | --- | --- | --- | --- |
|  | Mass fraction（%） | Atomic fraction（%） | Mass fraction（%） | Atomic fraction（%） |
| C | 39.01 | 45.88 | 52.46 | 58.65 |
| O | 49.42 | 43.63 | 32.67 | 27.42 |
| N | 9.48 | 9.56 | 14.27 | 13.68 |
| S | 2.10 | 0.93 | 0.61 | 0.25 |

**Note:** Because of more polycation inclined to absorbing the bottom of the PCOF membrane, the mass fraction and atomic fraction of N element were more than the top surface of the PCOF membrane. Conversely, the concentration of S element of upper surface was more than the lower surface, implying relatively more TpPa-SO_3_H components lied in the top. Thus, the distributions of PDDA and SCOF are different in the heterogeneous structure.

**Table S2.** The comparison of Li^+^/Mg^2+^ selectivity among commercial nanofiltration membranes, reported polymer-based nanofiltration membranes and this study.

| Membrane | Permeability (LMH∙bar^-1^) | S  Li^+^/Mg^2+^ | Feed concentration (ppm) | MgCl_2_/LiCl concentration ratio | Ref. |
| --- | --- | --- | --- | --- | --- |
| The commercial nanofiltration membranes | | | | | |
| Desal DK (GE Osmonics) | - | 3.2 | 4680/3520 | 18:1/22:1 | [8] |
| DK-1812 | - | 3.2 | 6000 | 40:1 (molar ratio) | [9] |
| NF90 (Filter twice) | - | 6.7 | 4000 | 56.7 | [10] |
| The polyamide-modified membranes | | | | | |
| DAPP/TMC/PAN | - | 2.6 | 2000 | 20:1 | [11] |
| PIP-MWCNTs/PEI/PES | 14 | 7.1 | 2000 | 21.4:1 | [12] |
| BPEI/TMC/EDTA | 0.6 | 9.2 | 2000 | 24:1 | [13] |
| PIP-TMC/PHF | 6.3 | 13.1 | 2000 | 21.4:1 | [14] |
| PES/CNC-COOH/PA NF | 4.2 | 12.2 | 2000 | 30:1 | [15] |
| (PES-GO)/PEI/TMC | 11.2 | 16.1 | 2000 | 20:1 | [16] |
| PIP-TMC/  [MimAP][Tf_2_N] | 4.7 | 8.1 | 2100 | 20:1 | [17] |
| PIP-TMC/  [MimAP][Tf_2_N] | 4.2 | 7.2 | 3100 | 30:1 | [17] |
| CQDs-NH_2_ NF | 12.0 | 14.4 | 2000 | 30:1 | [18] |
| PEI/GQDs-NH_2_/TMC | 12.0 | 27.9 | 2000 | 20:1 | [19] |
| QBPD Membrane | 16.1 | 5.9 | 2000 | 50:1 | [20] |
| Polyamide-TG-8 | 1.2 | 46.4 | 2000 | 50:1 | [21] |
| PEI/γ-CDs-TMC | 4.9 | 10.8 | 2000 | 30:1 | [22] |
| PEI-TMC/DABIL | 37.1 | 26.5 | 1000 (MgCl_2_) | 20:1 | [23] |
| RIP-0.250  membrane | 0.9 | 9.2 | 2000 | 20:1 | [24] |
| PEI@15C5-TMC | 8.0 | 11.9 | 2000 | 20:1 | [25] |
| Janus PEI/PIP-TMC | 10.6 | 18.3 | 2000 | 30:1 | [26] |
| This work | | | | | |
| The PCOF membrane | 4.7 | 58.2 | 2000 | 20:1 | This work |
| The PCOF membrane | 4.6 | 61.6 | 2000 | 50:1 |  |

**-:** not available

# Section S-8: Supplemental references

[1] a) J. S. da Silva, S. G. M. Carvalho, R. P. da Silva, A. C. Tavares, U. Schade, L. Puskar, F. C. Fonseca, B. R. Matos, *Phys. Chem. Chem. Phys.* **2020**, 22, 13764-13779; b) A. Lehmani, S. Durand-vidal, P. Turq, *J. Appl. Polym. Science* **1998**, *68,* 503-508.

[2] a) Y. Zhang, J. Guo, G. Han, Y. Bai, Q. Ge, J. Ma, C. H. Lau, L. Shao, *Sci. Adv.* **2021**, 7, eabe8706 ; b) Z. Guo, Y. Zhang, Y. Dong, J. Li, S. Li, P. Shao, X. Feng, B. Wang, *J. Am. Chem. Soc.* **2019**, 141, 1923-1927; c) L. Wang, C. Wang, Y. Ren, Z. Yang, Y. Zheng, Q. Zhang, W. Wu, J. Wang, *ACS Appl. Polym. Materials* **2023**, 5, 7562-7570; d) Z. Wang, Q. Yu, Y. Huang, H. An, Y. Zhao, Y. Feng, X. Li, X. Shi, J. Liang, F. Pan, P. Cheng, Y. Chen, S. Ma, Z. Zhang, *ACS Cent. Sci.* **2019**, 5, 1352-1359.

[3] N. Petzetakis, C. M. Doherty, A. W. Thornton, X. C. Chen, P. Cotanda, A. J. Hill, N. P. Balsara, *Nat. Commun.* **2015**, *6*, 7529.

[4] J. R. Maple, U. Dinur, A. T. Hagler, *Proc. Nati. Acad. Sci. U. S. A.* **1988**, *85*, 5350-5354.

[5] B. Pullman, Intermolecular forces: proceedings of the Fourteenth Jerusalem Symposium on Quantum Chemistry and Biochemistry held in Jerusalem, Israel, April, **1981**.

[6] a) P. Li, K. M. Merz, *J. Chem. Theory Comput.* **2013**, *10*, 289-297; b) P. Li, L. F. Song, K. M. Merz, *J. Chem. Theory Comput.* **2015**, *11*, 1645-1657.

[7] S. Plimpton, *J. Comput. Phys.* **1995**, 117, 1-19.

[8] G. Yang, H. Shi, W. Liu, W. Xing, N. Xu, *Chinese J. Chem. Eng.* **2011**, 19, 586-591.

[9] Q. Bi, Z. Zhang, C. Zhao, Z. Tao, *Water Sci. Technol.* **2014**, 70, 1690-1694.

[10] A. Somrani, A. H. Hamzaoui, M. Pontie, *Desalination* **2013**, 317, 184-192.

[11] X. Li, C. Zhang, S. Zhang, J. Li, B. He, Z. Cui, *Desalination* **2015**, 369, 26-36.

[12] H.-Z. Zhang, Z.-L. Xu, H. Ding, Y.-J. Tang, *Desalination* **2017**, 420, 158-166.

[13] W. Li, C. Shi, A. Zhou, X. He, Y. Sun, J. Zhang, *Sep. Purif. Technol.* **2017**, 186, 233-242.

[14] Q. Shen, S. J. Xu, Z. L. Xu, H. Z. Zhang, Z. Q. Dong, *J. Appl. Polym. Sci.* **2019**, 136, 48029.

[15] C. Guo, N. Li, X. Qian, J. Shi, M. Jing, K. Teng, Z. Xu, *Sep. Purif. Technol.* **2020**, 230, 115567.

[16] P. Xu, J. Hong, X. Qian, Z. Xu, H. Xia, Q.-Q. Ni, *Desalination* **2020**, 488, 114522.

[17] H. Wu, Y. Lin, W. Feng, T. Liu, L. Wang, H. Yao, X. Wang, *J. Membr. Sci.* **2020**, 603, 117997.

[18] C. Guo, X. Qian, F. Tian, N. Li, W. Wang, Z. Xu, S. Zhang, *Chem. Eng. J.* **2021**, 404, 127144.

[19] P. Xu, J. Hong, Z. Xu, H. Xia, Q.-Q. Ni, *Sep. Purif. Technol.* **2021**, 258, 118042.

[20] Y. Feng, H. Peng, Q. Zhao, *Sep. Purif. Technol.* **2022**, 280, 119848.

[21] S. Zhang, R. Zhang, R. Li, Z. Zhang, Y. Li, H. Deng, J. Zhao, T. Gu, M. Long, X. Wang, S. Zhang, Z. Jiang, *J. Membr. Sci.* **2022**, 663, 121063.

[22] Y. Zhao, N. Li, J. Shi, Y. Xia, B. Zhu, R. Shao, C. Min, Z. Xu, H. Deng, *Sep. Purif. Technol.* **2022**, 286, 120419.

[23] F. Soyekwo, H. Wen, D. Liao, C. Liu, *ACS Appl. Mater. Interfaces* **2022**, 14, 32420-32432. [24] Y. Li, S. Wang, W. Wu, H. Yu, R. Che, G. Kang, Y. Cao, *J. Membr. Sci.* **2022**, 659, 120809.

[25] H. Li, Y. Wang, T. Li, X.-K. Ren, J. Wang, Z. Wang, S. Zhao, *Chem. Eng. J.* **2022**, 438, 135658.

[26] C. Guo, Y. Qian, P. Liu, Q. Zhang, X. Zeng, Z. Xu, S. Zhang, N. Li, X. Qian, F. Yu, *ACS Appl. Mater. Interfaces* **2023**, 15, 4814-4825.
